# Pharmacological inhibition of noncanonical EED-EZH2 signaling overcomes chemoresistance in prostate cancer

**DOI:** 10.7150/thno.49235

**Published:** 2021-05-08

**Authors:** Xin Li, Lajos Gera, Shumin Zhang, Yanhua Chen, Lei Lou, Lauren Marie Wilson, Zhong-Ru Xie, Giuseppe Sautto, Degang Liu, Alira Danaher, Kenza Mamouni, Yang Yang, Yuhong Du, Haian Fu, Omer Kucuk, Adeboye O. Osunkoya, Jia Zhou, Daqing Wu

**Affiliations:** 1 Center for Cancer Research and Therapeutic Development and Department of Biological Sciences, Clark Atlanta University, Atlanta, GA, USA.; 2 Molecular Oncology and Biomarkers Program, Georgia Cancer Center; Department of Biochemistry and Molecular Biology, Medical College of Georgia, Augusta University, Augusta, GA, USA.; 3 Department of Biochemistry and Molecular Genetics, University of Colorado Denver, Anschutz Medical Campus, School of Medicine, Aurora, CO, USA.; 4 Department of Urology and Winship Cancer Institute, Emory University School of Medicine, Atlanta, GA, USA.; 5 Department of Hand Surgery, Union Hospital, Tongji Medical College, Huazhong University of Science and Technology, Wuhan, China.; 6 School of Electrical and Computer Engineering, College of Engineering, University of Georgia, Athens, GA, USA.; 7 Center for Vaccines and Immunology, University of Georgia, Athens, GA, USA.; 8 Sartorius Corporation, Bohemia, NY, USA.; 9 Department of Otorhinolaryngology, Union Hospital, Tongji Medical College, Huazhong University of Science and Technology, Wuhan, China.; 10 Department of Pharmacology and Chemical Biology, and Emory Chemical Biology Discovery Center, Emory University School of Medicine, Atlanta, GA, USA.; 11 Department of Hematology and Medical Oncology, Winship Cancer Institute, Emory University School of Medicine, Atlanta, GA, USA.; 12 Departments of Pathology and Laboratory Medicine, Emory University School of Medicine, Atlanta, GA, USA.; 13 Chemical Biology Program, Department of Pharmacology and Toxicology, University of Texas Medical Branch, Galveston, TX, USA.; 14 MetCure Therapeutics LLC, Atlanta, GA, USA.

**Keywords:** prostate cancer, chemoresistance, EED inhibitor, EZH2 signaling, drug discovery

## Abstract

**Rationale:** Chemoresistance is a major obstacle in prostate cancer (PCa) treatment. We sought to understand the underlying mechanism of PCa chemoresistance and discover new treatments to overcome docetaxel resistance.

**Methods:** We developed a novel phenotypic screening platform for the discovery of specific inhibitors of chemoresistant PCa cells. The mechanism of action of the lead compound was investigated using computational, molecular and cellular approaches. The *in vivo* toxicity and efficacy of the lead compound were evaluated in clinically-relevant animal models.

**Results:** We identified LG1980 as a lead compound that demonstrates high selectivity and potency against chemoresistant PCa cells. Mechanistically, LG1980 binds embryonic ectoderm development (EED), disrupts the interaction between EED and enhancer of zeste homolog 2 (EZH2), thereby inducing the protein degradation of EZH2 and inhibiting the phosphorylation and activity of EZH2. Consequently, LG1980 targets a survival signaling cascade consisting of signal transducer and activator of transcription 3 (Stat3), S-phase kinase-associated protein 2 (SKP2), ATP binding cassette B 1 (ABCB1) and survivin. As a lead compound, LG1980 is well tolerated in mice and effectively suppresses the *in vivo* growth of chemoresistant PCa and synergistically enhances the efficacy of docetaxel in xenograft models.

**Conclusions:** These results indicate that pharmacological inhibition of EED-EZH2 interaction is a novel strategy for the treatment of chemoresistant PCa. LG1980 and its analogues have the potential to be integrated into standard of care to improve clinical outcomes in PCa patients.

## Introduction

Prostate cancer (PCa) is the most commonly diagnosed cancer and the second leading cause of cancer death among American men 1. As the first-line chemotherapy for metastatic castration-resistant PCa, docetaxel initially prolongs overall survival by 3~4 months 2. However, most patients relapse and develop chemoresistance, eventually progressing without a cure 3.

Understanding the unique biological alterations in chemoresistant PCa cells is essential to the development of novel strategies to overcome docetaxel resistance. It has been proposed that multiple mechanisms contribute to chemoresistance 4. The rare subpopulations of cancer cells with stemness or neuroendocrine characteristics are considered as intrinsically drug-resistant, which can evade conventional therapies and result in recurrence and metastasis 5. Epithelial-to-mesenchymal transition (EMT), a major mechanism by which epithelial cancer cells gain invasive phenotypes, promotes self-renewal capability, increases expression of stem cell markers and confers chemoresistance 6-8. Among the mechanisms common to most cancer types, overexpression of membrane-bound drug efflux pumps (mainly ATP binding cassette B 1 [ABCB1), or multidrug resistance protein 1 [MDR1], p-glycoprotein) and anti-apoptotic proteins (such as survivin) have been frequently associated with therapeutic resistance in PCa 9-12. S-phase kinase-associated protein 2 (SKP2), the substrate recognition component of SCF (SKP1-CUL1-F-box) E3 ubiquitin-protein ligase complex, increases stem cell features and chemoresistance in PCa cells 13.

Enhancer of zeste homolog 2 (EZH2), a histone methyltransferase and a subunit of polycomb repressive complex 2 (PRC2), is commonly involved in transcriptional repression via PRC2-dependent histone 3 lysine 27 (H3K27) methylation 14, 15. EZH2 can also methylate non-histone protein substrates (such as signal transducer and activator of transcription 3, Stat3) and act as a co-activator for transcriptional factors including androgen receptor (AR), β-catenin and nuclear factor kappa B (NF-κB) 16-20. This noncanonical function may rely on EZH2 phosphorylation at Ser21 (p-EZH2[S21]). Interestingly, p-EZH2(S21) is significantly increased in clinical castration-resistant PCa 17.

EZH2 is overexpressed in a wide range of human cancers, therefore, it has been actively pursued as a prominent target for drug development 21, 22. A number of EZH2 inhibitors targeting the catalytic SET domain via competition with methyl-donating S-adenosylmethionine (SAM) have been developed and two of them have entered clinical trials 23-26. However, cancer cells manage to develop EZH2 mutations resistant to these SAM-competitive inhibitors, compromising the anticancer effects 27, 28. Recently, several novel strategies are being explored, including the destabilization of EZH2 protein and allosteric inhibition of PRC2 function 29-38, which could achieve a general and more efficient blockade of EZH2 oncogenic signaling in highly heterogeneous therapeutic-resistant tumors.

Embryonic ectoderm development (EED) protein is the “reader” component of the PRC2 complex, which binds trimethylated H3K27 (H3K27Me3) through the central pocket formed by seven WD-40 (β-transducin) repeats 14. The interaction between EED and EZH2 is required for the epigenetic “writer” function of EZH2 39, 40. Interrupting the EED-EZH2 complex, therefore, represents an attractive strategy for inhibiting EZH2 functions regardless of the mutation status of the EZH2 enzyme. Several small-molecule EED inhibitors have been developed, most binding to the central pocket and preventing allosteric activation of the catalytic activity of PRC2 29, 30, 33-37. MAK683, an EED inhibitor developed by Novartis, is the only one that has entered a Phase I/II clinical trial in lymphoma patients (NCT02900651).

Although the correlation between EZH2 overexpression and chemoresistance of human cancers or PCa progression has been reported 16, 17, 22, 41-44, the role of EZH2 in PCa chemoresistance remains to be further defined. In this study, we have uncovered a novel mechanism wherein a noncanonical EZH2 signal pathway consisting of Stat3, SKP2, ABCB1 and survivin confers chemoresistance in PCa cells. We further identified LG1980, a small-molecule EED inhibitor that demonstrates specific and potent *in vitro* and *in vivo* activities against chemoresistant PCa in preclinical models. As a lead compound, LG1980 exhibits potent anticancer activities and is well tolerated in animals. These results indicate that small-molecule inhibition of EED-EZH2 interaction is a promising strategy to overcome docetaxel resistance in PCa.

## Results

### A two-tier phenotypic screening platform for the discovery of selective inhibitors of chemoresistant PCa

**Figure 1:**
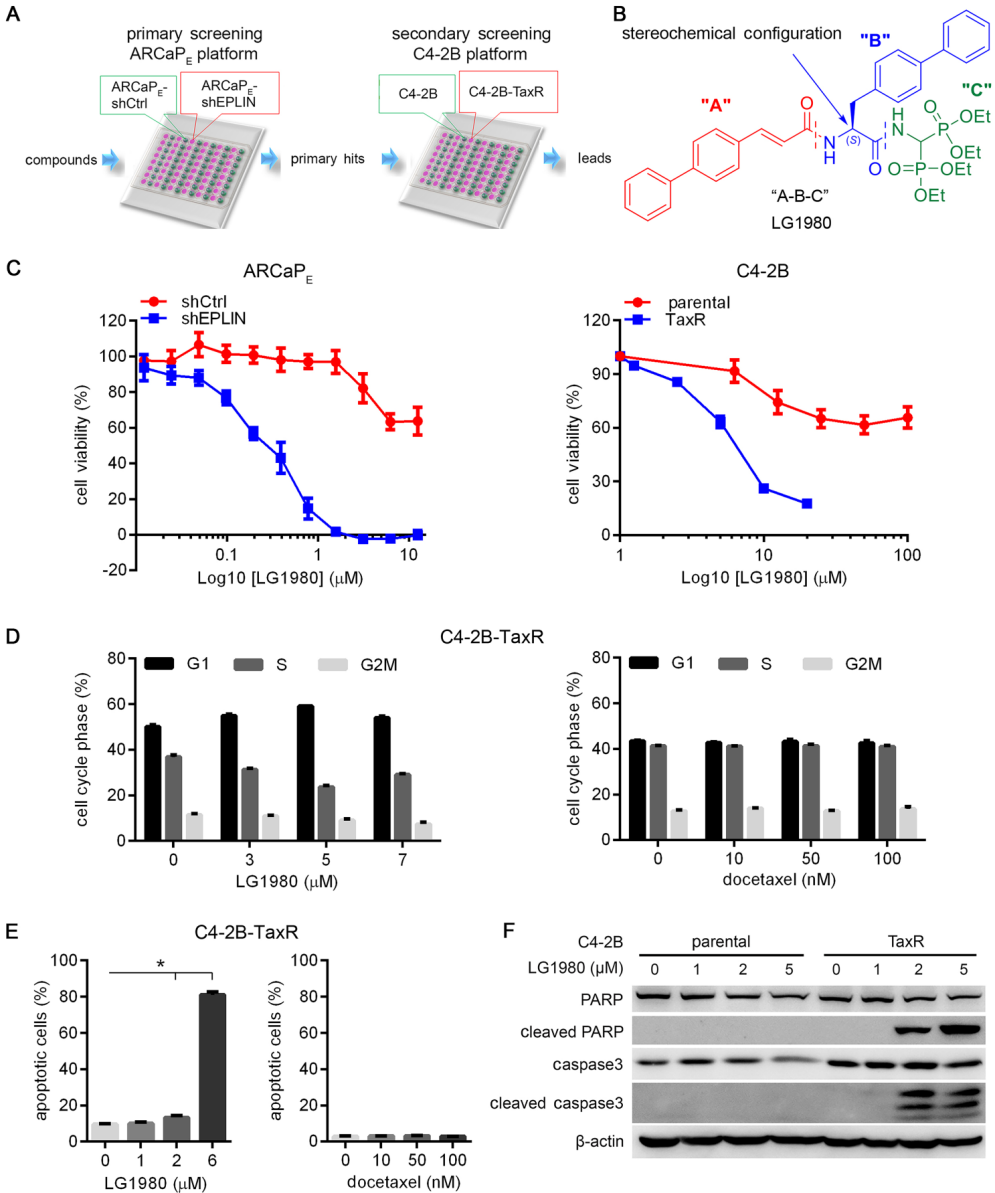
** LG1980 is a selective and potent inhibitor of chemoresistant PCa cells.** (A) A two-tier phenotypic screening platform for the discovery of inhibitors of chemoresistant PCa. Primary screening is performed to identify small-molecule compounds that selectively inhibit the *in vitro* viability of ARCaP_E_-shEPLIN but not ARCaP_E_-shCtrl cells. Primary hits are further screened in a second (orthogonal) assay for their potent *in vitro* cytotoxicity in C4-2B-TaxR cells, but not docetaxel-sensitive parental C4-2B cells. Potential leads are evaluated for their mechanism of action and *in vivo* efficacy against docetaxel-resistant PCa in xenograft models. (B) The “A-B-C” 3-component structure of LG1980. Note LG1980 is an “S”-stereoisomer. (C) *In vitro* cytotoxicity of LG1980 in the ARCaP_E_ and C4-2B models (72 h). (D) Left: Flow cytometry analysis of cell cycle in C4-2B-TaxR cells following LG1980 treatment at the indicated concentrations (48 h). *p* < 0.05 for all pairwise comparisons between the percentages of cells in each cell cycle from the control and LG1980 treatment groups, except those in G2M phase between the control and 3 μM LG1980-treated cells; Right: Flow cytometry analysis of cell cycle in C4-2B-TaxR cells following docetaxel treatment at the indicated concentrations (48 h). *p* > 0.05 for all pairwise comparisons between the percentages of cells in each cell cycle from the control and docetaxel treatment groups. (E) Left: Flow cytometry analysis on Annexin V staining in C4-2B-TaxR cells following LG1980 treatment at the indicated concentrations (72 h). *p < 0.05; Right: Flow cytometry analysis on Annexin V staining in C4-2B-TaxR cells following docetaxel treatment at the indicated concentrations (72 h). *p* > 0.05 for all pairwise comparisons between the control and different concentrations of docetaxel. (F) Western bot analysis on the expression of apoptotic markers in C4-2B and C4-2B-TaxR cells following LG1980 treatment at the indicated concentrations (72 h). β-actin was used as the loading control.

Both intrinsic and acquired mechanisms contribute to chemoresistance, particularly in advanced cancers. To increase the success rates of discovering more effective drug candidates, an excellent screening system is expected to recapitulate the high heterogeneity of chemoresistant tumors. Our phenotypic system consists of a sequential screening in two independent cellular models of chemoresistant PCa, i.e., ARCaP_E_-shEPLIN 45 and C4-2B-TaxR 9 (Figure 1A). The rationale for selecting these models are: (1) Previously we demonstrated that epithelial protein lost in neoplasm (EPLIN), a key molecule in the maintenance of epithelial structure at adherens junctions, is a suppressor of metastasis in PCa and other solid tumors 45-47. EPLIN knockdown in low-invasive PCa cells (such as ARCaP_E_) 48, 49 promotes EMT, enhances invasiveness, increases the expression of stem cell markers and confers chemoresistance. Compared with the control cells (ARCaP_E_-shCtrl), EPLIN-depleted ARCaP_E_ cells (ARCaP_E_-shEPLIN) are more resistant to the treatment of common chemotherapeutics, including docetaxel 45 (Figure S1A). These results indicated that ARCaP_E_-shEPLIN cells could represent a subpopulation of stem-like cancer cells that are intrinsically chemoresistant, and potential inhibitors of chemoresistant PCa could be identified based on their capability of selectively targeting ARCaP_E_-shEPLIN cells in differential viability assays; (2) To confirm primary hits and eliminate false positives from the ARCaP_E_-shEPLIN/ARCaP_E_-shCtrl platform, we further included an orthogonal assay consisting of C4-2B, an AR-positive and bone metastatic PCa line 50, and C4-2B-TaxR, a highly docetaxel-resistant C4-2B derivative that represents the phenotypes of acquired chemoresistance 9 (Figure S1B). Only the primary hits that also demonstrated potent cytotoxicity in C4-2B-TaxR cells, but not in parental C4-2B cells, would be considered as potential leads for further evaluation; (3) the two models are also different in the activation status of AR signaling. ARCaP_E_ cells are androgen-repressive whereas C4-2B cells maintain high androgen responsiveness 51, 52. Taken together, this two-tier screening platform could recapitulate the heterogeneity and complex biology of advanced PCa, increase hit rate, reduce false positives and maximize the capabilities of identifying specific inhibitors of chemoresistant PCa.

### LG1980 is a selective and potent inhibitor of chemoresistant PCa cells

Using a ”molecular hybridization” strategy, we have developed several generations of peptidomimetic compounds with a 3-component “A-B-C” structure, where “A”, “B” and “C” represent different pharmacophoric moieties 53-55. In the case of LG1980, for instance, the “A” component is a cinnamic acid derivative, the “B” component is a biphenyl amino acid residue and the “C” component is an aminomethylenebisphosphonate moiety (Figure 1B). We screened a panel of newly developed compounds for their selective cytotoxicity in chemoresistant ARCaP_E_-shEPLIN and C4-2B-TaxR cells (Table 1, Figure S2). Among the tested compounds, LG1980 potently inhibited the *in vitro* viability of ARCaP_E_-shEPLIN cells (IC_50_ = 0.26 µM), but exhibited weak cytotoxicity in ARCaP_E_-shCtrl cells (IC_50_ = 16.88 µM). The selectivity index (SI) of LG1980, defined as the ratio of the IC_50_ of LG1980 in ARCaP_E_-shCtrl and that in ARCaP_E_-shEPLIN cells, was determined as 64.9. Consistently, LG1980 displayed an IC_50_ of 6.87 µM in C4-B-TaxR cells but a much higher IC_50_ (91.12 µM) in parental C4-2B cells, with an SI of 13.2 (Figure 1C). Flow cytometry showed that LG1980 effectively inhibited cell cycle progression at the G1/S checkpoint (Figure 1D, left; Figure S3A) and induced apoptosis (Figure 1E, left; Figure S3B) in C4-2B-TaxR cells. As a control, docetaxel did not cause any significant changes to the cell cycle progression and apoptosis in these cells (Figure 1D, 1E, right panels; Figure S3A, S3B). Western blotting analyses further confirmed that LG1980 induced the cleavage of poly (ADP-ribose) polymerase (PARP) and caspase-3 in C4-2B-TaxR cells in a dose-dependent manner. In comparison, LG1980 treatment at the same concentrations did not significantly activate apoptosis in parental C4-2B cells (Figure 1F). Consistently, LG1980 effectively induced apoptosis in a dose-dependent manner in ARCaP_E_-shEPLN cells (Figure S3C). Taken together, these results indicated that LG1980 is a selective and potent inhibitor of chemoresistant PCa cells.

### LG1980 is a novel EED inhibitor

**Figure 2:**
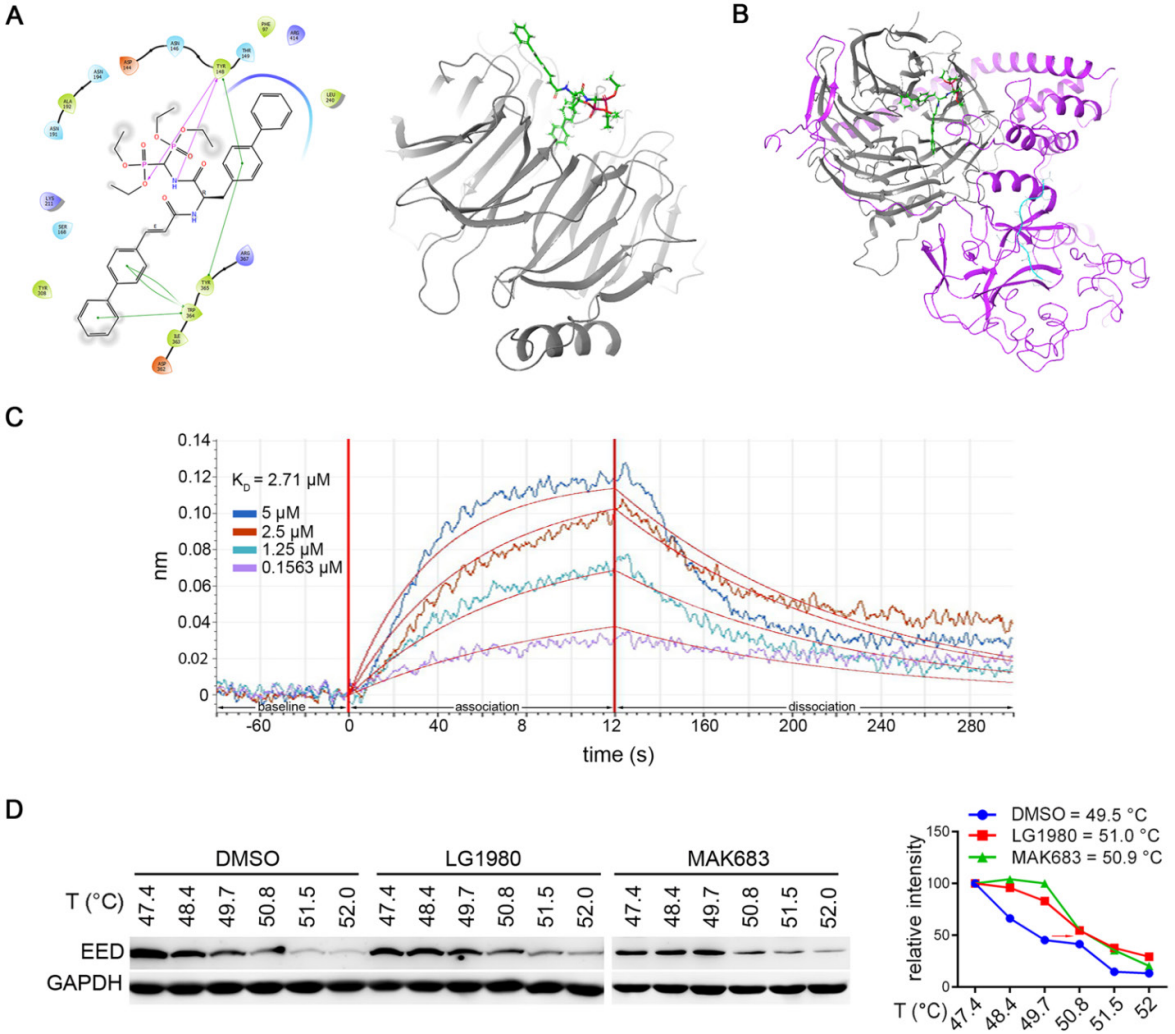
** LG1980 is a novel EED inhibitor.** (A) Left: key amino acid residues and bonds mediating the interaction between LG1980 and EED. Pink lines refer to hydrogen bonds and the green lines refer to pi-pi bonds; Right: docked structure of LG1980 (green) and EED protein (gray). (B) Docked structure of LG1980 (green) and the EED (gray)-EZH2 (purple) complex. The binding of H3K27Me3 (blue) is also shown. (C) Binding of LG1980 to randomly biotinylated EED protein on ForteBio Octet Red384 System. X-axis, time in seconds; Y-axis, binding in nm. R^2^ was calculated as 0.9202. (D) Left: CETSA analysis of EED expression in C4-2B-TaxR cells treated with DMSO or LG1980 (50 µM, 1 h). MAK683 (10 µM, 1 h) was used as a positive control. Right: melting temperature curves of EED protein in the presence of DMSO, LG1980 or MAK683 in C4-2B-TaxR cells.

Molecular docking analyses identified LG1980 as a novel pharmacological inhibitor of EED. Specifically, LG1980 bound the central pocket of human EED protein and docked to the side opposite to the EZH2 structure. The ligand interaction diagram showed that one of the diphenyl groups was angled inwards in the binding pocket, and had interactions with Trp364, Tyr365 and Tyr148. There were also interactions between both an oxygen, a nitrogen and Tyr148 (Figure 2A). Interestingly, when EZH2 was introduced into the docking model (Figure 2B), the binding affinity of LG1980 to the EED-EZH2 complex (-33.69) became significantly lower than that of EED only (-77.16) (Table 2). These results suggested that LG1980 preferably bound EED and it might interrupt the interactions between EED and EZH2 to achieve a more stable LG1980-EED conformation.

The *in vitro* binding affinity between LG1980 and EED was evaluated by biolayer interferometry using the FortéBio Octet RED384 system. Recombinant human EED protein was randomly biotinylated and used for the binding assay. As shown in Figure 2C, LG1980 could directly bind EED protein in a dose-dependent manner, with an equilibrium dissociation constant (*K_D_*) value of 2.71 μM. The *in vivo* binding of LG1980 and EED was further confirmed in C4-2B-TaxR cells using cellular thermal shift assay (CETSA), a technique widely used for the validation of drug target engagement in live cells 56-60. The results in Figure 2D demonstrated that upon LG1980 treatment, there was a thermal stabilization of EED protein, as evidenced by a shift in melting temperature (*Tm*). As the positive control, MAK683 induced a similar degree of shift in *Tm* of EED protein in C4-2B-TaxR cells. Taken together, these computational and experimental results indicated that LG1980 is a specific EED inhibitor.

### LG1980 disassembles the PRC2 complex and induces the degradation of key PRC2 components in chemoresistant PCa cells

**Figure 3:**
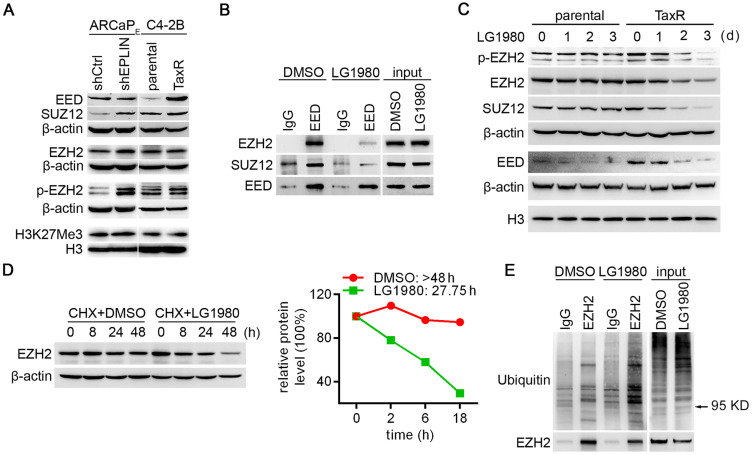
** LG1980 disassembles the PRC2 complex and destabilizes EZH2 protein in chemoresistant PCa cells.** (A) Protein expression of core PRC2 components in the ARCaP_E_ and C4-2B models. (B) Western bot analysis on the expression of EZH2 and SUZ12 in IgG- and EED antibody-immunoprecipitates in C4-2B-TaxR cells treated with LG1980 or vehicle control (7 µM, 16 h). (C) Western bot analysis on the expression of p-EZH2, EZH2, SUZ12 and EED in C4-2B and C4-2B-TaxR cells treated with LG1980 (7 µM) at the indicated time points. Both β-actin and histone H3 were used as loading controls. (D) Left: Expression of EZH2 in C4-2B-TaxR cells treated with DMSO or LG1980 (7 µM) in the presence of CHX; Right: Calculated half-life of EZH2 protein in C4-2B-TaxR cells treated with DMSO or LG1980 (7 µM). (E) Western bot analysis on the expression of polyubiquitination in IgG- and EZH2 antibody-immunoprecipitates in C4-2B-TaxR cells treated with LG1980 or vehicle control (7 µM, 16 h). Arrow indicates the approximate size of EZH2 protein.

The stability of EZH2 protein and its catalytic activity as a histone methyltransferase require the presence of at least two other PRC2 core subunits, i.e., EED and SUZ12 14, 61-63, therefore we examined protein expression profile of EZH2, EED and SUZ12 in the ARCaP_E_-shCtrl/ARCaP_E_-shEPLIN and C4-2B/C4-2B-TaxR models (Figure 3A). All three components are expressed in the two pairs of PCa cells, with the levels of EZH2 slightly higher in ARCaP_E_-shEPLIN and unchanged in C4-2B-TaxR, EED unchanged in ARCaP_E_-shEPLIN and higher in C4-2B-TaxR, SUZ12 higher in ARCaP_E_-shEPLIN and unchanged in C4-2B-TaxR. H3K27Me3, an indicator of canonical EZH2 function, was highly expressed in all the cells examined, indicating an active PRC2 signaling. On the other hand, however, there was no significant difference in H3K27Me3 levels between chemoresistant sublines and their parental counterparts, suggesting that the canonical EZH2 signaling may not play a major role in chemoresistance. Intriguingly, p-EZH2(S21) was markedly increased in both ARCaP_E_-shEPLIN and C4-2B-TaxR cells. These results pointed to the possibility that a noncanonical EZH2 signaling may be associated with chemoresistance.

We determined whether LG1980 affected EED interaction with EZH2 and SUZ12. The C4-2B-TaxR subline was used as the primary model, since it closely mimics the clinicopathological features of AR-positive, chemoresistant and bone-metastatic PCa 9, 64. C4-2B-TaxR cells were treated with LG1980 or the vehicle for 16 h prior to immunoprecipitation using an EED or IgG antibody. As shown in Figure 3B, LG1980 significantly reduced EED-associated EZH2 and SUZ12 without reducing the expression of EED, EZH2 or SUZ12 in the total cell lysates. These results indicated that LG1980 may interrupt the interactions between EED, EZH2 and SUZ12 and effectively disassemble the PRC2 complex.

EED and SUZ12 are the limiting factors for PRC2 complex formation, which is required for the stabilization of EZH2 protein 61-63. Several recent studies using specific EED-targeted inhibitors found that interrupting EED interaction with the PRC2 complex could induce significant degradation of EZH2, EED, and SUZ12 29, 30, 38. In consistence with these observations, LG1980 effectively inhibited the expression of EZH2, p-EZH2(S21), EED and SUZ12 in C4-2B-TaxR cells in a time-dependent manner (Figure 3C). In comparison, LG1980 only decreased EED level but did not markedly affect the expression of EZH2, p-EZH2(S21) or SUZ12 in parental C4-2B cells.

We investigated the mechanism by which LG1980 reduced the expression of these key PRC2 components. At the mRNA level, LG1980 didn't significantly affect the expression of *EZH2* (Figure S4A), suggesting that the effect of LG1980 on EZH2 expression may primarily occur at post-translational levels. Indeed, in the presence of cycloheximide (CHX), an inhibitor of *de novo* protein synthesis, LG1980 significantly shortened the half-life (T_1/2_) of EZH2 protein from > 48.0 h to 27.8 h (Figure 3D). Similarly, LG1980 reduced the calculated T_1/2_ of EED from 7.3 h to 4.0 h, and the T_1/2_ of SUZ12 from > 18.0 h to 9.4 h (Figure S4B). We further performed EZH2 immunoprecipitation in C4-2B-TaxR cells treated with LG1980 or the vehicle for 16 h, then examined the expression of ubiquitinated EZH2. As shown in Figure 3E, LG1980 treatment significantly increased the levels of EZH2-associated polyubiquitination, suggesting that LG1980-induced EZH2 downregulation may be mediated by proteasome-dependent degradation.

Taken together, these results supported a mechanism of action wherein LG1980 effectively induced the disassembly of the PRC2 complex and promoted the degradation of key PRC2 components, subsequently reducing the expression of EZH2 and p-EZH2(S21) in chemoresistant PCa cells.

### A noncanonical EZH2 signaling activates Stat3 and upregulates SKP2, ABCB1 and survivin in chemoresistant PCa cells

**Figure 4:**
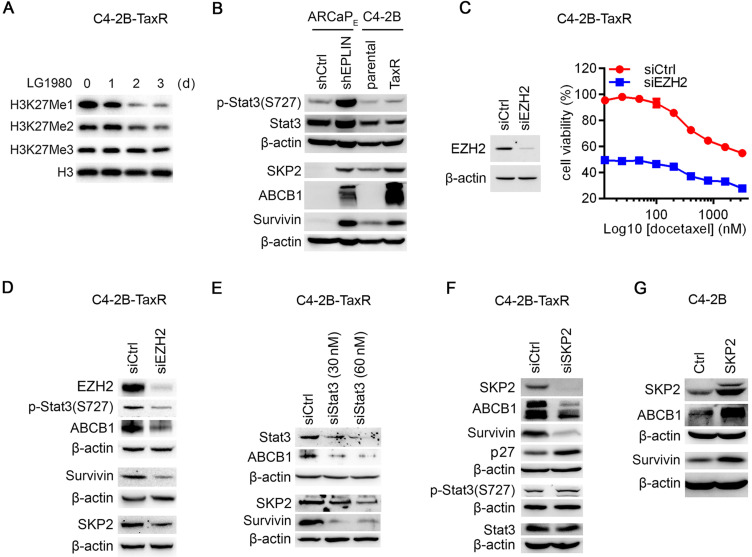
** EZH2 regulates noncanonical Stat3-SKP2-ABCB1/survivin survival signaling in chemoresistant PCa cells.** (A) Western blot analysis on the expression of H3K27 methylation in C4-2B-TaxR cells treated with LG1980 (7 µM) at the indicated time points. (B) Protein expression of p-Stat3, Stat3, SKP2, ABCB1 and survivin in the ARCaP_E_ and C4-2B models. (C) Left: Western blot analysis on the expression of EZH2 in C4-2B-TaxR cells transfected with control or EZH2 siRNA (60 nM, 72 h); Right: *In vitro* viability of C4-2B-TaxR cells transfected with control siRNA or EZH2 siRNA (60 nM, 72 h) and in the presence of varying concentrations of docetaxel. (D) Protein expression of EZH2, p-Stat3, SKP2, ABCB1 and survivin in C4-2B-TaxR cells transfected with control or EZH2 siRNA (60 nM, 72 h). (E) Western blot analysis on the expression of Stat3, SKP2, ABCB1 and survivin in C4-2B-TaxR cells transfected with control or Stat3 siRNA (72 h). (F) Western blot analysis on the expression of p-Stat3, Stat3, SKP2, ABCB1, survivin and p27 in C4-2B-TaxR cells transfected with control or SKP2 siRNA (60 nM, 72 h). (G) Western blot analysis on the expression of SKP2, ABCB1 and survivin in C4-2B cells transfected with SKP2 expression vector or control plasmid (72 h).

An interesting observation was that, although LG1980 significantly reduced the expression of EZH2 and p-EZH2(S21) (Figure 3C), it only significantly inhibited the mono- and di-methylation of H3K27, but to a much lesser degree, the tri-methylation, an indicator of canonical EZH2 function (Figure 4A). Consistently, LG1980 did not significantly affect the expression of known EZH2 target genes, such as *CASP1* and *RTP4*, in C4-2B-TaxR cells (Figure S4C). These results indicated that LG1980 may mainly interfere with a noncanonical function of EZH2 in chemoresistant PCa cells.

The elevated expression of p-EZH2(S21) (Figure 3A) and selective inhibition of p-EZH2(S21) by LG1980 (Figure 3C) in chemoresistant PCa cells provided an important clue to understanding the mechanism of action of LG1980. Previous studies have demonstrated that phosphorylation of EZH2 at Ser21 selectively reduces its affinity for H3K27me3 without affecting the ability of EZH2 to bind other PRC2 component, making EZH2 more available to non-histone substrates, such as Stat3 19, 20. EZH2 activates Stat3 methylation at lysine residues, resulting in Stat3 phosphorylation and subsequent Stat3-dependent gene transcription. Significantly, this p-EZH2(S21)-dependent Stat3 activation occurs preferentially in stem-like glioblastoma cells and not in non-stem cancer cells 19. Activation of Stat3 signaling has further been shown to upregulate the expression of multiple oncogenic proteins involved in chemoresistance, including SKP2 and survivin 9-13, 65, 66. Therefore, we examined the expression profile of these oncogenic factors in the ARCaP_E_ and C4-2B models (Figure 4B). In consistent with our previous observations 67, p-Stat3(S727) was significantly increased in both ARCaP_E_-shEPLIN and C4-2B-TaxR cells. The expression of SKP2, survivin and interestingly, ABCB1, was also markedly increased in chemoresistance PCa cells.

**Figure 5:**
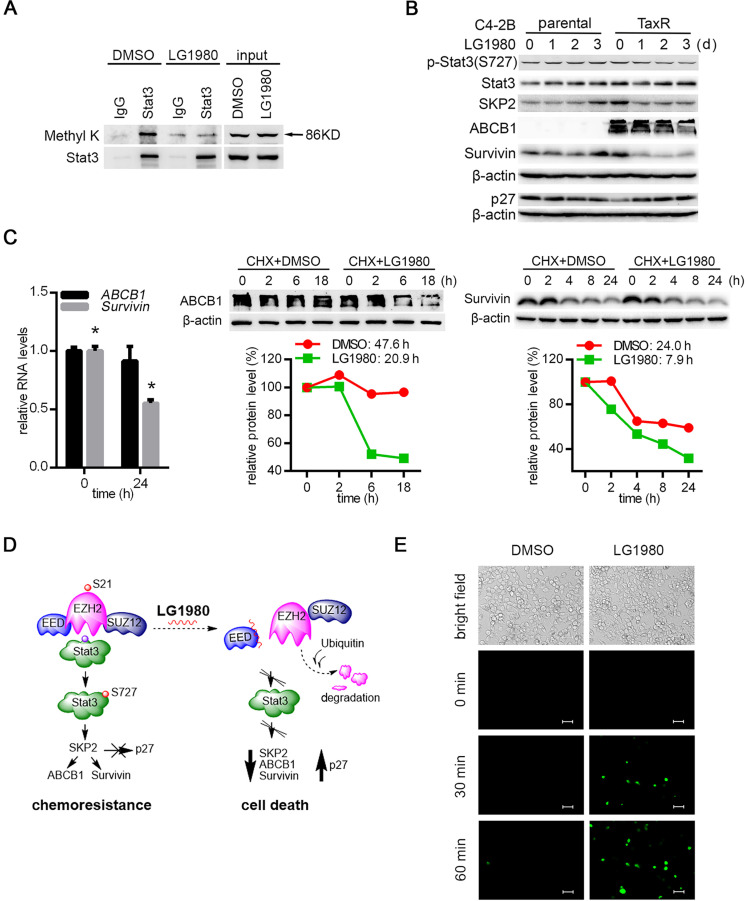
** LG1980 inhibits the noncanonical EZH2-Stat3-SKP2-ABCB1/survivin signaling in chemoresistant PCa cells.** (A) Western blot analysis of lysine methylation in the IgG- or Stat3 antibody-immunoprecipitates in C4-2B-TaxR cells treated with DMSO or LG1980 (7 µM, 24 h). (B) Protein expression of p-Stat3, Stat3, SKP2, ABCB1, survivin and p27 in C4-2B and C4-2B-TaxR cells following LG1980 treatment (7 µM) at the indicated time points. (C) Left: qPCR analysis of RNA expression of *ABCB1* and *survivin* in C4-2B-TaxR cells treated with LG1980 (7 µM, 24 h). * *p* < 0.01. Middle and right: C4-2B-TaxR cells were pre-incubated with CHX (50 µg/mL, 2h) prior to the treatment with DMSO or LG1980 (7 µM) for the indicated times. Protein expression of ABCB1 and survivin was analyzed by Western blotting and quantitated using the ImageJ program. (D) A proposed mechanism of action of LG1980. In chemoresistant PCa cells, EZH2 phosphorylation initiates noncanonical signaling via the methylation and phosphorylation of non-histone substrate Stat3, thereby activating the expression of SKP2, ABCB1 and survivin and inhibiting p27. LG1980 binds EED, disrupts EED-EZH2 interaction and causes ubiquitin-mediated degradation of EZH2, thereby reducing p-EZH2 and suppressing Stat3-dependent survival signals. These events eventually induce apoptosis in chemoresistant PCa cells and sensitize them to chemotherapeutics. (E) Fluorescence microscopy images of cellular uptake of Oregon Green 488-paclitaxel in C4-2B-TaxR cells. Cells were first treated with LG1980 (7 µM) for 72 h prior to paclitaxel incubation for the indicated times. Scale bar: 50 µm.

We determined the functional connections between these signaling molecules in chemoresistant PCa cells: (1) EZH2 depletion significantly inhibited the *in vitro* viability of C4-2B-TaxR cells by approximately 48.5%. In the presence of varying concentrations of docetaxel, EZH2 knockdown also resulted in a significant decrease in viable C4-2B-TaxR cells (Figure 4C). These results indicated that EZH2 is required for the maintenance of chemoresistance in PCa cells; (2) EZH2 knockdown in C4-2B-TaxR cells effectively inhibited the phosphorylation of Stat3 at S727, suggesting that EZH2 is required for the activation of Stat3 signaling. EZH2 depletion also resulted in the concurrent downregulation of SKP2, survivin and ABCB1 (Figure 4D). In comparison, EZH2 depletion only significantly inhibited the expression of H3K27Me1 and H3K27Me2, but to a much lesser degree, of H3K27Me3 (Figure S4D), an effect that was similar to that of LG1980 treatment (Figure 4A); (3) siRNA depletion of Stat3 in C4-2B-TaxR cells resulted in a marked reduction of SKP2 and survivin. ABCB1 level was also decreased in the Stat3 siRNA-transfected cells, indicating that ABCB1 is under the control of Stat3 signaling (Figure 4E); (4) SKP2 depletion decreased the expression of ABCB1 and survivin and increased p27, a known SKP2 downstream target 68. In comparison, SKP2 knockdown did not affect the expression of p-Stat3(S727) or total Stat3 (Figure 4F); (5) Consistently, ectopic expression of SKP2 in parental C4-2B cells increased the expression of ABCB1 and survivin (Figure 4G). Taken together, these results indicated that a noncanonical EZH2 signaling can activate Stat3 and upregulate SKP2, ABCB1 and survivin in chemoresistant PCa cells.

### LG1980 inhibits noncanonical EZH2-Stat3-SKP2-ABCB1/survivin signaling in chemoresistant PCa cells

Since LG1980 selectively inhibited p-EZH2(S21) in chemoresistant PCa cells, presumably by promoting EZH2 protein degradation, we investigated the hypothesis that LG1980 might target the noncanonical EZH pathway consisting of Stat3, SKP2, ABCB1and survivin: (1) Immunoprecipitation-Western blot analyses showed that in the Stat3 immunoprecipitates from C4-2B-TaxR cells, LG1980 significantly inhibited Stat3 methylation at lysines (Figure 5A), suggesting that LG1980 may affect Stat3 signaling via the blockade of Stat3 methylation 19; (2) LG1980 effectively inhibited the expression of p-Stat3(S727), SKP2, ABCB1 and survivin and increased the expression of p27 in C4-2B-TaxR cells. In comparison, LG1980 did not significantly affect the expression of these proteins in parental C4-2B cells (Figure 5B). At the mRNA level, LG1980 reduced *survivin* expression by approximately 50%, but had negligible effect on *ABCB1* expression in C4-2B-TaxR cells (Figure 5C, left). These data indicated that LG1980 may mainly inhibit ABCB1 expression at post-transcriptional levels, and affect survivin expression at both transcriptional and post-translational levels. To test this hypothesis, C4-2B-TaxR cells were incubated in the presence of CHX prior to the treatment with LG1980 or vehicle control. As shown in Figure 5C, LG1980 reduced the half-life (T_1/2_) of ABCB1 protein from 47.6 h to 20.9 h and the T_1/2_ of survivin protein from 24.0 h to 7.9 h. Furthermore, in C4-2B-TaxR cells pre-incubated with a proteasome inhibitor MG132, LG1980 significantly increased the accumulation of ubiquitinated proteins with corresponding molecular weights of ABCB1 and survivin (Figure S5A). These results suggested that LG1980 may promote protein degradation of ABCB1 and survivin via a proteasome-ubiquitin-dependent mechanism.

Taken together, these mechanistic studies supported a working model that LG1980 may interrupt the EED-EZH2 interaction, disassemble the PRC2 complex, destabilize core PRC2 components (EED, EZH2, and SUZ12) and reduce the level of p-EZH2(S21), thereby selectively inhibiting the noncanonical EZH2-Stat3-SKP2-ABCB1/survivin signaling and inducing apoptosis in chemoresistant PCa cells (Figure 5D).

### LG1980-mediated ABCB1 downregulation increases paclitaxel uptake in chemoresistant PCa cells

Overexpression of the transmembrane pump ABCB1 is a major mechanism for multi-drug resistance in many cancer types 10. We examined whether the LG1980 inhibition on ABCB1 expression can affect the cellular uptake of chemotherapeutics. Pre-treatment with LG1980 resulted in a rapid (within 30 min) accumulation of fluorescence dye (Oregon Green 488)-conjugated paclitaxel in C4-2B-TaxR cells. In comparison, there was no paclitaxel uptake until 60 min in the control cells (Figure 5E). We further determined the effect of ABCB1 silencing on chemoresistance in C4-2B-TaxR cells. As shown in Figure S5B, ABCB1 depletion with siRNAs significantly increased the intracellular presence of Oregon Green 488-paclitaxel. These results indicated that LG1980-mediated ABCB1 downregulation could be responsible for the increased uptake and intracellular retention of chemotherapeutics in chemoresistant PCa cells.

### *In vitro* and* in vivo* safety profile of LG1980

**Figure 6:**
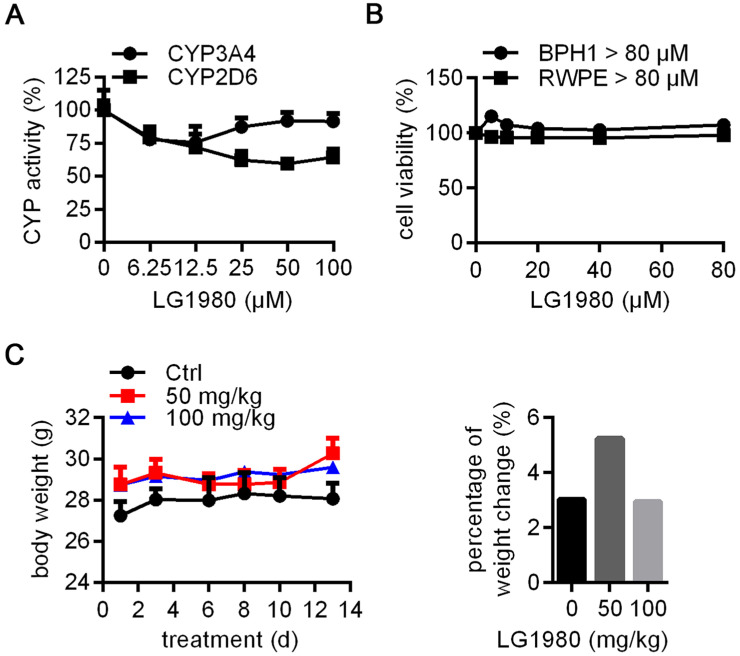
***In vitro* and *in vivo* safety profile of LG1980.** (A) *In vitro* effect of LG1980 on the activities of CYP450 3A4 and 2D6. (B) CCK-8 assay of the *in vitro* cytotoxicity of LG1980 in BPH-1 and RWPE-1 cells (72 h). (C) Left: Average body weights of healthy CD-1 mice treated with vehicle (n = 3), LG1980 (50 mg/kg, n = 3) or LG1980 (100 mg/kg, n = 4) via subcutaneous route, three times per week; Right: Percentage of mouse body weight change in different treatment groups.

Inhibition of cytochrome P450 (CYP450) metabolic enzymes is a major cause of clinical toxicity and drug withdrawal 69. Particularly, CYP 3A4 and 2D6 are the two major determinants of the metabolism of most clinical drugs, including docetaxel 70-74. As shown in Figure 6A, LG1980 had very weak inhibition on the activities of 3A4 and 2D6, even when used at high concentrations. These results indicated that LG1980 exhibits weak drug-drug interaction 69 and is unlikely to affect the bioavailability of co-administered chemotherapeutics, such as docetaxel.

Cell culture studies showed that LG1980 (up to 80 µM) did not affect the *in vitro* proliferation of human prostatic epithelial cells RWPE-1 and BPH1 (Figure 6B), indicating low cytotoxicity to normal/benign cells. The ineffectiveness of LG1980 in such cells may be attributed to the lack of an intact and functional PRC2 complex, for example, very low expression of EED (Figure S6). We further evaluated the repeat-dose sub-chronic toxicity of LG1980 in healthy CD-1 mice via subcutaneous injection, a clinically-relevant route. Compared with the vehicle control, the administration of LG1980 at two high doses, i.e., 50 mg/kg and 100 mg/kg, three times per week, did not have any significant side effects in animals, as demonstrated by their normal body weight gains and behaviors in all groups (Figure 6C). *Ex vivo* examination of major organs did not observe any abnormalities (Table S1).

### As a monotherapy, LG1980 inhibits the skeletal growth of chemoresistant C4-2B-TaxR tumors

**Figure 7:**
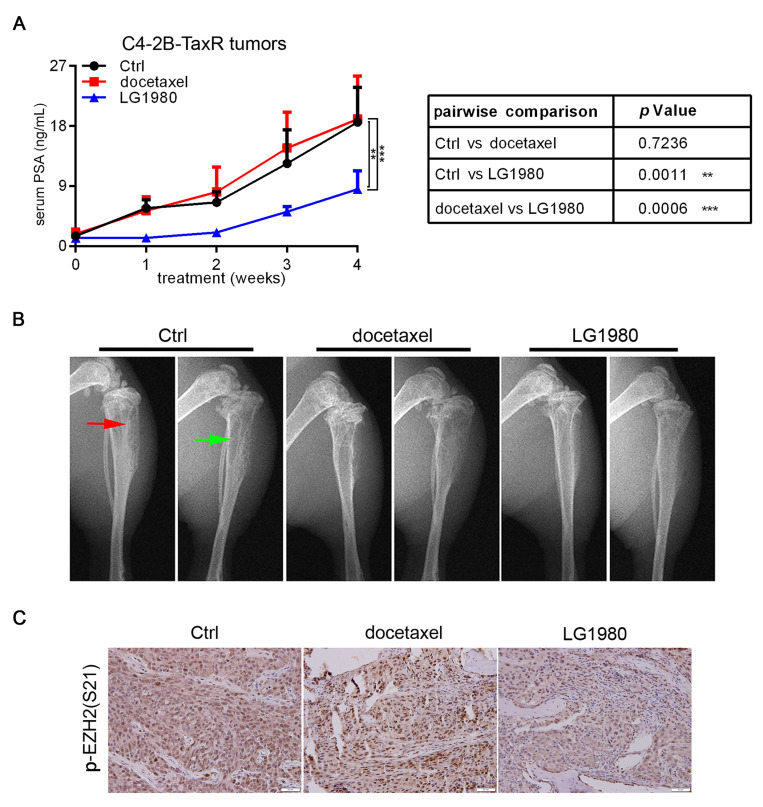
** As a monotherapy, LG1980 inhibits the *in vivo* growth of chemoresistant PCa and enhances docetaxel efficacy in athymic nude mice.** (A) Left: serum PSA values of C4-2B-TaxR xenograft-bearing mice treated with vehicle (n = 4), docetaxel (5 mg/kg, i.p, once per week; n = 3), or LG1980 (20 mg/kg, i.p, three times per week; n = 5); Right: two-way ANOVA analysis of the PSA values between different treatment groups. ** *p* < 0.01, *** *p* < 0.001. (B) Representative x-ray radiography of tumor-bearing mouse tibias in different treatment groups. Red arrow: osteoblastic lesions; green arrow: osteolytic lesions. (C) IHC expression of p-EZH2(S21) in C4-2B-TaxR bone tumor tissues. Scale bar: 50 µm.

As a single agent, LG1980 selectively and effectively inhibited the *in vitro* viability of chemoresistant PCa cells (Figure 1C). We evaluated the *in vivo* efficacy of LG1980 against bone-metastatic and chemoresistant PCa in the intratibial C4-2B-TaxR xenografts. Previous studies have shown that serum prostate-specific antigen (PSA) value is a reliable, quantitative indicator of the *in vivo* growth of PCa xenografts, particularly in mouse bones 75-83. Following a 4-week treatment, intraperitoneal injection of LG1980 (20 mg/kg, 3 times per week) significantly reduced the average PSA level when compared with either the vehicle control or docetaxel treatment. In contrast, there was no statistical difference in the PSA levels between the control group and docetaxel-treated mice (Figure 7A). X-ray radiography showed that LG1980 treatment was associated with reduced osteolytic lesions and improved architecture in tumor-bearing tibias (Figure 7B). Immunohistochemistry demonstrated that compared with the vehicle control or docetaxel, LG1980 effectively reduced the tissue levels of p-EZH2(S21) in C4-2B-TaxR bone tumors (Figure 7C). LG1980 treatment was not associated with significant changes in the average body weights of tumor-bearing mice (Figure S7A). Taken together, these results indicated that as a monotherapy, LG1980 is efficacious in retarding the skeletal growth of chemoresistant PCa.

### As an adjunct therapy, LG1980 enhances the efficacy of docetaxel against the skeletal growth of C4-2 xenografts

**Figure 8:**
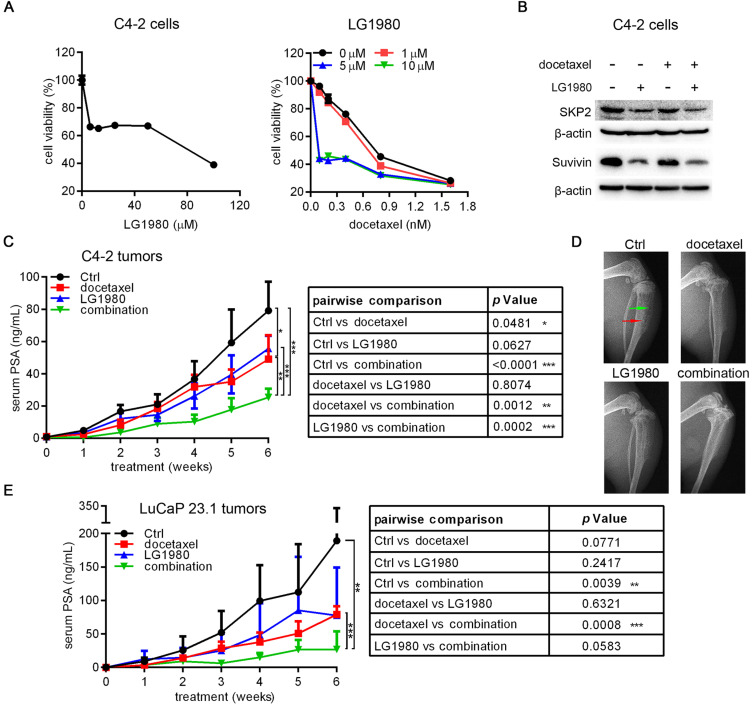
** As an adjunct therapy, LG1980 synergistically enhances the *in vivo* efficacy of docetaxel against the skeletal growth of C4-2 tumors and the subcutaneous growth of LuCaP 23.1 tumors in athymic nude mice.** (A) Left: *In vitro* cytotoxicity of LG1980 in C4-2 cells (72 h); Right: *In vitro* cytotoxicity of docetaxel in C4-2 cells in the presence of varying concentrations of LG1980 (72 h). (B) Western blot analysis of protein expression of SKP2 and survivin in C4-2 cells treated with LG1980 and docetaxel. (C) Left: serum PSA values of C4-2-Luc tumor-bearing mice treated with vehicle control (n = 5), docetaxel (5 mg/kg, i.p, once per week; n = 5), LG1980 (10 mg/kg, i.p, three times per week; n = 5), or the combination of docetaxel and LG1980 (n = 5). Right: two-way ANOVA analysis of the PSA values between different treatment groups. * *p* < 0.05, ** *p* < 0.01, *** *p* < 0.001. (D) Representative x-ray radiography of tumor-bearing mouse tibias in different treatment groups. Red arrow: osteoblastic lesions; green arrow: osteolytic lesions. (E) Left: serum PSA values of LuCaP 23.1 tumor-bearing mice treated with vehicle (n = 3), docetaxel (5 mg/kg, once per week; n = 5), LG1980 (20 mg/kg, three times per week; n = 5), or the combination of docetaxel and LG1980 (n = 6). Right: two-way ANOVA analysis of the PSA values between different groups. ** *p* < 0.01, *** *p* < 0.001.

A unique feature of LG1980 is that it demonstrates high selectivity against chemoresistant PCa cells, but only having weak cytotoxicity in chemoresponsive cells (Figure 1C and Table 1). Consistently, LG1980 had a high IC_50_ of 56.06 µM in C4-2 cells, an androgen-independent and docetaxel-responsive LNCaP derivative 50 (Figure 8A, left). Interestingly, the addition of low-concentration LG1980 in C4-2 cultures significantly augmented the *in vitro* cytotoxicity of docetaxel. For example, the IC_50_ of docetaxel was reduced from 1.94 nM to 0.39 nM (a 4.9-fold decrease) in the presence of 5 µM LG1980, demonstrating a strong synergy between the two agents (Figure 8A, right). At the molecular levels, the combination of LG1980 (5 µM) and docetaxel (0.5 nM) reduced the expression of SKP2 and survivin (Figure 8B). These molecular changes may contribute to the synergistic cytotoxicity of the combination regimen.

We evaluated the *in vivo* efficacy of the combination of LG1980 and docetaxel in athymic nude mice carrying intratibial C4-2-Luc tumors. Mice were treated for 6 weeks with low-dose LG1980 (10 mg/kg, three times per week), docetaxel (5 mg/kg, once per week), the combination of LG1980 and docetaxel, or vehicle, respectively, via the intraperitoneal route. Compared with the control group, docetaxel treatment had a moderate inhibitory effect on tumor growth, but LG1980 monotherapy did not significantly reduced PSA level. Importantly, there were significant differences in the PSA values between the combination group and the vehicle control, docetaxel or LG1980 alone groups, respectively (Figure 8C). X-ray radiography demonstrated improved bone structure in mice treated with the combination regimen (Figure 8D). LG1980 treatment was not associated with a significant reduction of body weights of tumor-bearing mice (Figure S7B). These results demonstrated that at low doses, LG1980 synergistically enhanced the *in vivo* efficacy of docetaxel against the skeletal growth of C4-2 xenografts.

### The combination of LG1980 and docetaxel inhibits the *in vivo* growth of LuCaP 23.1 xenograft tumors

We further evaluated the *in vivo* efficacy of LG1980 as a monotherapy or in combination with docetaxel in LuCaP 23.1 tumors, a patient-derived xenograft (PDX) model established from PCa metastases and resistant to docetaxel treatment 84. Athymic nude mice bearing subcutaneous LuCaP 23.1 tumors at both flanks were randomized and treated for 6 weeks with docetaxel (5 mg/kg, once a week), LG1980 (20 mg/kg, 3 times per week), the combination of LG1980 and docetaxel, or vehicle, respectively, via intraperitoneal injection. Compared with the control group, the treatment with either docetaxel or LG1980 alone did not significantly suppress the growth of LuCaP 23.1 tumors. In contrast, the combination of LG1980 and docetaxel significantly retarded tumor growth when compared with the control or docetaxel groups (Figure 8E). These results indicated that the combination of LG1980 and docetaxel was effective in suppressing the *in vivo* growth of PCa. LG1980 treatment was not associated with significant changes in the average body weights of LuCP 23.1 tumor-bearing mice (Figure S7C).

## Discussion

Herein, we established a phenotypic screening platform for the discovery of small-molecule inhibitors of chemoresistant PCa. We identified LG1980 as a new EED inhibitor that demonstrated high specificity and potency against chemoresistant PCa cells. We described a novel mechanism of chemoresistance wherein EZH2 phosphorylation activates a noncanonical EZH2 oncogenic signaling. LG1980 exerts its anticancer activity by affecting the PRC2 complex assembly and inducing protein degradation of key PRC2 components, including EZH2, subsequently reducing the expression of p-EZH2(S21) and inhibiting noncanonical EZH2 signaling (Figure 5D). LG1980 was well tolerated in animals and exhibited acceptable safety pharmacology profiles. As a monotherapy, LG1980 effectively inhibited the skeletal growth of chemoresistant C4-2B-TaxR tumors; and as an adjunct agent, LG1980 enhanced the *in vivo* efficacy of docetaxel against C4-2 and LuCaP23.1 xenografts. These results indicated that pharmacological inhibition of the noncanonical EED-EZH2 signaling is a promising strategy to overcome PCa chemoresistance. To our knowledge, LG1980 is a first-in-class selective inhibitor of chemoresistant PCa, which has the potential of being integrated into standard of care to improve clinical outcomes.

The success of hypothesis-driven, target-based drug discovery depends on the validation of the physiological importance of the target in a specific disease. While progress has been made in understanding the mechanism of chemoresistance, these efforts have been significantly limited by the high heterogeneity and complex biology of advanced cancer 4. As a noticeable consequence, efficient drug screening platforms are still lacking for the discovery of specific inhibitors of chemoresistant PCa. In this study, we have utilized a combinational approach by performing target deconvolution studies following sequential phenotypic screens in two independent cellular models, i.e, the ARCaP_E_-shEPLIN/ARCaPE-shCtrl and C4-2B-TaxR/C4-2B pairs. These two models differ significantly with regard to their mechanism of resistance and molecular characteristics: ARCaP_E_-shEPLIN represents a subpopulation of PCa cells that gain stemness and exhibit intrinsic chemoresistance via EMT 6-8, 45, whereas C4-2B-TaxR cells mimic the clinical progression of acquired docetaxel resistance 9. Despite these distinct differences, both chemoresistant sublines seem to rely on a convergent EED-EZH2-Stat3-SKP2-ABCB1/survivin signaling cascade to survive and escape docetaxel chemotherapy. Selective inhibition of this survival mechanism can provide an effective approach to eliminate chemoresistance PCa cells whereas sparing chemosensitive cancer cells or normal cells. Supporting this notion, LG1980 demonstrates high selectivity against chemoresistant PCa cells and exhibits an excellent *in vitro* and *in vivo* safety profile.

The canonical function of EZH2 is to catalyze the mono-, di-, and tri-methylation of H3K27, thereby acting as a transcriptional repressor in a variety of physiological and pathological processes 14. Interestingly, there was no significant difference in the expression of H3K27Me3 between chemoresistant PCa cells and their chemoresponsive counterparts (Figure 3A), indicating that the canonical EZH2 signaling may not play an important role in chemoresistance. Supporting this notion, three highly selective, SAM-competitive EZH2 inhibitors (CPI-1205, GSK126 and EPZ-6438) had much higher IC_50_ values in chemoresistant PCa cells and did not affect major components of the putative noncanonical EZH2 signaling (Figure S8). On the other hand, EZH2 can act as a transcriptional activator of multiple oncogenes in a PRC2-independent manner, promoting tumor progression and therapeutic resistance 16-19. Of particular interest, EZH2 phosphorylation at Ser21 only reduces the affinity of EZH2 toward H3 histone without compromising PRC2 composition, thus allowing the binding and lysine methylation of non-histone substrates, such as Stat3 20. For example, EZH2 phosphorylation at Ser21 and the EZH2-Stat3 interaction preferentially occur in stem-like glioblastoma relative to non-stem bulk tumor cells, subsequently activating Stat3 phosphorylation and Stat3-dependent transcription 19, 20. This functional connection between p-EZH2(S21) and Stat3 activation was also observed in chemoresistant PCa cells, indicating that EZH2 may have a noncanonical role in the activation of Stat3 signaling and the acquisition of chemoresistance, which is independent of the epigenetic repressor activity of EZH2. Our results from the two independent and diverse cellular models suggested that chemoresistant PCa cells may rely on, or become “addicted to”, the noncanonical EED-EZH2-Stat3-SKP2-ABCB1/survivin signaling to survive and escape standard chemotherapy. Therefore, targeting this pivotal survival mechanism may provide a promising approach to overcome chemoresistance and eliminate lethal PCa cells.

An interesting observation from the current study is that although LG1980 reduced the expression of EZH2, it did not have a significant effect on the canonical function of EZH2, as evidenced by unchanged expression of H3K27Me3 and known EZH2 target genes (Figure 4A, Figure S4C). In fact, EZH2-containing PRC2 complex is not the sole histone methyltransferase responsible for methylation on H3K27 and transcriptional silencing. For example, EZH1, a sequence homolog of EZH2, can form homodimers (EZH1/EZH1) or heterodimers (EZH1/ZEH2) to maintain H3K27Me3 level and prevent the derepression of PRC2 target genes 40, 85. EZH1 and EZH2 are functionally redundant in the slowly proliferating precursors of malignant peripheral nerve sheath tumor, and the depletion of EZH2 from these cells (such as ipNF05.5) only partially affects H3K27 trimethylation 86. Bortezomib, a proteasome inhibitor for the treatment of multiple myeloma, significantly reduced EZH2 (but not EZH1) in MM.1S myeloma cells and did not affect H3K27Me3 expression 87. Currently there is no study on the role of EZH1 in H3K27 methylation in PCa cells, but it would be plausible to postulate that when EZH2 is phosphorylated at Ser21 and preferentially associated with Stat3 in chemoresistant PCa cells, EZH1 (and possibly other histone methyltransferases) can complement the canonical EZH2 function and maintain the H3K27Me3 mark. In fact, LG1980 only selectively inhibited the noncanonical EZH2-Stat3 signaling but slightly increased EZH1 expression in C4-2B-TaxR cells (Figure S9), which may provide an explanation for the ineffectiveness of LG1980 on canonical EZH2 signaling.

MAK683 is the only EED inhibitor currently under clinical evaluation. Although MAK683 demonstrated similar molecular effects on the expression of p-EZH2, p-Stat3(S727), SKP2, ABCB1 and survivin with LG1980 in C4-2B-TaxR cells (Figure S10A), it had similar IC_50_ values in parental C4-2B (9.44 µM) and chemoresistant C4-2B-TaxR (6.69 µM) cell, respectively, with an SI of 1.4 (Figure S10B). Furthermore, MAK683 appeared to be more cytotoxic than LG1980 in normal/benign prostatic epithelial cells, with an IC_50_ of 25.47 µM and 20.69 µM in RWPE-1 and BPH1 cells, respectively (Figure S10C; note the IC_50_ of LG1980 in both cell lines were > 80 µM, Figure 6B). Although these results were only from immortalized human cell lines, they suggested that LG1980 is potentially less toxic *in vivo*, as supported by the repeat-dose toxicity studies (Figure 6C).

In summary, our investigation into the mechanism of action of LG1980 has uncovered a novel function of EED-EZH2 interaction in the regulation of PCa chemoresistance. These findings not only validated the therapeutic significance of EED-EZH2-Stat3-SKP2-ABCB1/survivin signaling, but also demonstrated LG1980 as a novel EED inhibitor that potently induces apoptosis in chemoresistant PCa cells. Furthermore, by promoting EZH2 protein degradation, LG1980 may be more effective than SAM-competitive EZH2 inhibitors in targeting PRC2 functions in highly heterogeneous, therapeutic-resistant tumors (Figure S8A). As we recently reported, LG1980 has a high retention and slow clearance rate in rat plasma, suggesting the compound is sufficiently stable in circulation 88, which could be an advantage of using LG1980 in targeting metastatic cancer cells. Although certain pharmaceutical properties of LG1980 are still suboptimal and need to be improved 88, LG1980 demonstrates a high selectivity against chemoresistant PCa with a satisfactory safety profile. Continued development of LG1980 and its analogues as a novel class of inhibitors of chemoresistant PCa is warranted.

## Materials and Methods

### Chemical Synthesis

LG1980 (Pcin-Bip-AMDP(OEt_4_), C_39_H_46_N_2_O_8_P_2_: 732.75 dalton) was synthesized using a two-step procedure as we described previously 55 (Figure S11). LG1980 was purified by preparative reversed-phase high-performance liquid chromatography (RP-HPLC). The purity and homogeneity of final products were cross-checked by analytical RP-HPLC and liquid chromatography-mass spectrometry.

### Molecular Docking

The structures of human EED protein (PDB ID:5wuk, resolution: 2.03 Å) 89 and EED-EZH2 complex (PDB ID: 5HYN resolution: 2.0 Å) were obtained from the Protein Data Bank 90. The proteins were prepared using the “protein preparation” wizard of Schrödinger Maestro. The SMILES (Simplified Molecular Input Line Entry System) of LG1980 is CP(=O)(C)C(NC(=O)[C@H](Cc1ccc(cc1)c2ccccc2)NC(=O)/C=C/c3ccc(cc3)c4ccccc4)P(=O)(C)C. The conformations of LG1980 and MAK683 were generated using “LigPrep” module of Maestro. The potential binding sites predicted by Schrödinger “SiteMap” module and LISE server 91 were the same as the binding site of bound ligand on 5wuk. The receptor grid was generated at the predicted binding site using “Glide” module of Maestro and docking was conducted in extra precision (XP) docking protocol.

### Cell Culture and Chemicals

Human PCa ARCaP_E_ cells stably expressing human EPLIN short hairpin RNA (shRNA) (ARCaP_E_-shEPLIN) or control shRNA (ARCaP_E_-shCtrl) were established and cultured as we described previously 45. C4-2 cells (provided by Dr. Leland WK Chung at Cedars-Sinai Medical Center) were routinely cultured in T-medium (Life Technologies, Carlsbad, CA) supplemented with 5% fetal bovine serum (FBS; Atlanta Biologicals, Atlanta, GA) and penicillin-streptomycin (Corning, Corning, NY). C4-2B and its docetaxel-resistant derivative C4-2B-TaxR (provided by Dr. Allen C. Gao at University of California Davis) were cultured following the procedures described in 9, with the modification that C4-2B-TaxR cells were routinely maintained in the presence of 100 nM docetaxel (LC Laboratories, Woburn, MA). The final concentration of docetaxel in culture medium was reduced to 5 nM before experimental assays. Human prostate epithelial cell lines RWPE-1 (provided by Dr. Ruoxiang Wang at Cedars-Sinai Medical Center) and BPH-1 (provided by Dr. Balakrishna Lokeshwar at Georgia Cancer Center) were cultured following the procedures described in 92 and 93, respectively. Cell Counting Kit-8 (CCK-8; Dojindo Molecular Technologies, Inc., Rockville, MD) following the manufacturer's instruction. The half minimal inhibitory concentration (IC_50_) of specified agent was calculated with SigmaPlot program (Systat Software Inc., San Jose, CA). Cycloheximide (CHX), dimethyl sulfoxide (DMSO), MG132, propidium iodide were purchased from Sigma-Aldrich (St. Louis, MO). MAK683 was obtained from Biovision Inc (Milpitas, CA).

CPI-1205, GSK126 and EPZ-6438 were purchased from Sellechchem Chemicals LLC (Houston, TX).

### *In vitro* Binding Affinity Measurement

Biolayer interferometry analysis on a ForteBio Octet Red384 instrument (Sartorius BioAnalytical Instruments, Inc., Fremont, CA) was carried out using biotinylated EED protein (Novus Biologicals, Centennial, CO). The biotinylated EED (30 µg/mL) was loaded onto super streptavidin sensors in 50 µL loading buffer (PBS + 0.02% Tween-20) for 10 min resulting in a 14 nm loading density. Subsequently, the biosensors were quenched in 20 µg/mL biocytin for 2 min and washed in the loading buffer for 3 min. EED-loaded biosensors were then pre-equilibrated in kinetic buffer (cat. No. 18-1105, Sartorius BioAnalytical Instruments, Inc., Fremont, CA) supplemented with 1% DMSO for 180 s. The same buffer was used throughout the rest of the assay. The kinetics of compound association were monitored by dipping biosensors into wells containing compound at different concentrations for 120 s. This was followed by dissociation in buffer for an additional 180 s. Reference biosensors were subjected to the same experimental procedures except that loading was done in loading buffer without biotinylated EED, which would be used to measure non-specific binding (NSB) of compound to biosensors. Binding curves were produced by the subtraction of NSB from the sensorgram of EED-loaded biosensors. The data was analyzed with Fortebio Data Analysis HT software (v12).

### *In vivo* Xenograft Models

All animal procedures were performed in compliance with Augusta University Institutional Animal Care and Use Committee (IACUC) and National Institutes of Health guidelines. A total of 2×10^6^ C4-2-Luc or C4-2B-TaxR cells were inoculated into the bilateral tibia of male athymic nude mouse (5-week old, Envigo RMS, Inc, Indianapolis, IN). Following the confirmation of tumor formation by rising PSA levels in mouse sera, mice were randomized into different groups and treated with vehicle (100% DMSO), LG1980, docetaxel or the combination at the indicated doses and schedules via intraperitoneal route. Mice were weighed twice per week, and tumor growth in bilateral tibia was monitored by serum PSA once a week using an enzyme-linked immunosorbent assay (ELISA) kit from United Biotech, Inc (Mountain View, CA). At the end point, X-ray radiography was performed using MX-20 System (Faxitron, Tucson, Arizona). At the end point, tumor-bearing tibias were harvested and fixed in 10% neutralized formalin for future analysis.

Human PCa patient-derived xenograft (PDX) strain LuCaP 23.1 84 was provided by Dr. Eva Corey (University of Washington) and routinely passaged subcutaneously in male *C.B-17* SCID mice (Charles River Laboratories Inc, Wilmington, MA) or athymic nude mice. For treatment experiments, LuCaP 23.1 tumors were harvested when the diameter reached 1 cm, cut into small pieces with the size of approximate 2 × 2 × 2 mm^3^, then implanted subcutaneously into both flanks of male athymic nude mouse (5-week old). Following the confirmation of tumor formation by rising PSA levels in mouse sera, mice were randomly divided into 4 groups and treated with vehicle, docetaxel, LG1980 or the combination of LG1980 and docetaxel at the indicated doses and schedules, respectively, via intraperitoneal injection. Mice were weighed twice per week, and tumor growth was monitored by serum PSA analysis. At the end point, subcutaneous tumors were harvested and fixed in 10% neutralized formalin for future analysis.

### Statistical Analysis

All *in vitro* data represent three or more experiments. The unpaired *t*-test was used to determine the significance of differences between any two groups. Two-way analysis of variance (ANOVA) was used to test the overall difference between different treatment groups during the whole study period. Prism 7.03 program (GraphPad Software Inc., La Jolla, CA) was used to perform the statistical analyses. *p* < 0.05 was considered as statistically significant.

## Supplementary Material

Supplementary figures and tables.

## Figures and Tables

**Table 1:** IC_50_ (µM) and SI of representative LG compounds in the ARCAP_E_ and C4-2B models

Compound	LG1980	LG1800	LG2134	LG2136
ARCAP_E_-shCtrl	16.88	0.75	0.75	0.25
ARCAP_E_-shEPLIN	0.26	0.25	0.5	0.25
SI* in ARCAP_E_ model	64.9	3.0	1.5	1.0
C4-2B	91.12	7.66	>20.0	>10.0
C4-2B TaxR	6.87	>10.0	>20.0	>20.0
SI in C4-2B model	13.2	<1.0	ND**	ND

*: selectivity index; **: not determined.

**Table 2:** Predicted binding affinity of LG1980 on EED protein and EED-EZH2 complex

Protein(s)	EED only	EED-EZH2 complex
Binding Affinity	-77.16	-33.69

## References

[1] Siegel RL, Miller KD, Jemal A (2020). Cancer statistics, 2020. CA Cancer J Clin.

[2] Tannock IF, de Wit R, Berry WR, Horti J, Pluzanska A, Chi KN (2004). Docetaxel plus prednisone or mitoxantrone plus prednisone for advanced prostate cancer. N Engl J Med.

[3] Quinn DI, Sandler HM, Horvath LG, Goldkorn A, Eastham JA (2017). The evolution of chemotherapy for the treatment of prostate cancer. Annals of oncology: official journal of the European Society for Medical Oncology.

[4] Galletti G, Leach BI, Lam L, Tagawa ST (2017). Mechanisms of resistance to systemic therapy in metastatic castration-resistant prostate cancer. Cancer Treat Rev.

[5] Wade CA, Kyprianou N (2018). Profiling Prostate Cancer Therapeutic Resistance. Int J Mol Sci.

[6] Marquardt S, Solanki M, Spitschak A, Vera J, Putzer BM (2018). Emerging functional markers for cancer stem cell-based therapies: Understanding signaling networks for targeting metastasis. Semin Cancer Biol.

[7] Fischer KR, Durrans A, Lee S, Sheng J, Li F, Wong ST (2015). Epithelial-to-mesenchymal transition is not required for lung metastasis but contributes to chemoresistance. Nature.

[8] Zheng X, Carstens JL, Kim J, Scheible M, Kaye J, Sugimoto H (2015). Epithelial-to-mesenchymal transition is dispensable for metastasis but induces chemoresistance in pancreatic cancer. Nature.

[9] Zhu Y, Liu C, Nadiminty N, Lou W, Tummala R, Evans CP (2013). Inhibition of ABCB1 expression overcomes acquired docetaxel resistance in prostate cancer. Mol Cancer Ther.

[10] Gottesman MM, Fojo T, Bates SE (2002). Multidrug resistance in cancer: role of ATP-dependent transporters. Nat Rev Cancer.

[11] Zhang M, Mukherjee N, Bermudez RS, Latham DE, Delaney MA, Zietman AL (2005). Adenovirus-mediated inhibition of survivin expression sensitizes human prostate cancer cells to paclitaxel *in vitro* and *in vivo*. Prostate.

[12] Zhang M, Latham DE, Delaney MA, Chakravarti A (2005). Survivin mediates resistance to antiandrogen therapy in prostate cancer. Oncogene.

[13] Ruan D, He J, Li CF, Lee HJ, Liu J, Lin HK (2017). Skp2 deficiency restricts the progression and stem cell features of castration-resistant prostate cancer by destabilizing Twist. Oncogene.

[14] Moritz LE, Trievel RC (2018). Structure, mechanism, and regulation of polycomb-repressive complex 2. J Biol Chem.

[15] Cao R, Wang L, Wang H, Xia L, Erdjument-Bromage H, Tempst P (2002). Role of histone H3 lysine 27 methylation in Polycomb-group silencing. Science.

[16] Yu J, Yu J, Mani RS, Cao Q, Brenner CJ, Cao X (2010). An integrated network of androgen receptor, polycomb, and TMPRSS2-ERG gene fusions in prostate cancer progression. Cancer Cell.

[17] Xu K, Wu ZJ, Groner AC, He HH, Cai C, Lis RT (2012). EZH2 oncogenic activity in castration-resistant prostate cancer cells is Polycomb-independent. Science.

[18] Min J, Zaslavsky A, Fedele G, McLaughlin SK, Reczek EE, De Raedt T (2010). An oncogene-tumor suppressor cascade drives metastatic prostate cancer by coordinately activating Ras and nuclear factor-kappaB. Nat Med.

[19] Kim E, Kim M, Woo DH, Shin Y, Shin J, Chang N (2013). Phosphorylation of EZH2 activates STAT3 signaling via STAT3 methylation and promotes tumorigenicity of glioblastoma stem-like cells. Cancer Cell.

[20] Cha TL, Zhou BP, Xia W, Wu Y, Yang CC, Chen CT (2005). Akt-mediated phosphorylation of EZH2 suppresses methylation of lysine 27 in histone H3. Science.

[21] Chase A, Cross NC (2011). Aberrations of EZH2 in cancer. Clin Cancer Res.

[22] Varambally S, Dhanasekaran SM, Zhou M, Barrette TR, Kumar-Sinha C, Sanda MG (2002). The polycomb group protein EZH2 is involved in progression of prostate cancer. Nature.

[23] Garapaty-Rao S, Nasveschuk C, Gagnon A, Chan EY, Sandy P, Busby J (2013). Identification of EZH2 and EZH1 small molecule inhibitors with selective impact on diffuse large B cell lymphoma cell growth. Chem Biol.

[24] Knutson SK, Wigle TJ, Warholic NM, Sneeringer CJ, Allain CJ, Klaus CR (2012). A selective inhibitor of EZH2 blocks H3K27 methylation and kills mutant lymphoma cells. Nat Chem Biol.

[25] Konze KD, Ma A, Li F, Barsyte-Lovejoy D, Parton T, Macnevin CJ (2013). An orally bioavailable chemical probe of the Lysine Methyltransferases EZH2 and EZH1. ACS Chem Biol.

[26] Stazi G, Zwergel C, Mai A, Valente S (2017). EZH2 inhibitors: a patent review (2014-2016). Expert Opin Ther Pat.

[27] Gibaja V, Shen F, Harari J, Korn J, Ruddy D, Saenz-Vash V (2016). Development of secondary mutations in wild-type and mutant EZH2 alleles cooperates to confer resistance to EZH2 inhibitors. Oncogene.

[28] Baker T, Nerle S, Pritchard J, Zhao B, Rivera VM, Garner A (2015). Acquisition of a single EZH2 D1 domain mutation confers acquired resistance to EZH2-targeted inhibitors. Oncotarget.

[29] Potjewyd F, Turner AW, Beri J, Rectenwald JM, Norris-Drouin JL, Cholensky SH (2020). Degradation of Polycomb Repressive Complex 2 with an EED-Targeted Bivalent Chemical Degrader. Cell Chem Biol.

[30] Hsu JH, Rasmusson T, Robinson J, Pachl F, Read J, Kawatkar S (2020). EED-Targeted PROTACs Degrade EED, EZH2, and SUZ12 in the PRC2 Complex. Cell Chem Biol.

[31] Ma A, Stratikopoulos E, Park KS, Wei J, Martin TC, Yang X (2020). Discovery of a first-in-class EZH2 selective degrader. Nat Chem Biol.

[32] Wang X, Cao W, Zhang J, Yan M, Xu Q, Wu X (2017). A covalently bound inhibitor triggers EZH2 degradation through CHIP-mediated ubiquitination. EMBO J.

[33] He Y, Selvaraju S, Curtin ML, Jakob CG, Zhu H, Comess KM (2017). The EED protein-protein interaction inhibitor A-395 inactivates the PRC2 complex. Nat Chem Biol.

[34] Huang Y, Zhang J, Yu Z, Zhang H, Wang Y, Lingel A (2017). Discovery of First-in-Class, Potent, and Orally Bioavailable Embryonic Ectoderm Development (EED) Inhibitor with Robust Anticancer Efficacy. J Med Chem.

[35] Li L, Zhang H, Zhang M, Zhao M, Feng L, Luo X (2017). Discovery and Molecular Basis of a Diverse Set of Polycomb Repressive Complex 2 Inhibitors Recognition by EED. PLoS One.

[36] Qi W, Zhao K, Gu J, Huang Y, Wang Y, Zhang H (2017). An allosteric PRC2 inhibitor targeting the H3K27me3 binding pocket of EED. Nat Chem Biol.

[37] Kong X, Chen L, Jiao L, Jiang X, Lian F, Lu J (2014). Astemizole arrests the proliferation of cancer cells by disrupting the EZH2-EED interaction of polycomb repressive complex 2. J Med Chem.

[38] Martin MC, Zeng G, Yu J, Schiltz GE (2020). Small Molecule Approaches for Targeting the Polycomb Repressive Complex 2 (PRC2) in Cancer. J Med Chem.

[39] Denisenko O, Shnyreva M, Suzuki H, Bomsztyk K (1998). Point mutations in the WD40 domain of Eed block its interaction with Ezh2. Mol Cell Biol.

[40] Yu JR, Lee CH, Oksuz O, Stafford JM, Reinberg D (2019). PRC2 is high maintenance. Genes Dev.

[41] Qiu Z, Zhu W, Meng H, Tong L, Li X, Luo P (2019). CDYL promotes the chemoresistance of small cell lung cancer by regulating H3K27 trimethylation at the CDKN1C promoter. Theranostics.

[42] Li P, Zhang X, Wang H, Wang L, Liu T, Du L (2017). MALAT1 is associated with poor response to oxaliplatin-based chemotherapy in colorectal cancer patients and promotes chemoresistance through EZH2. Molecular cancer therapeutics.

[43] Ougolkov AV, Bilim VN, Billadeau DD (2008). Regulation of pancreatic tumor cell proliferation and chemoresistance by the histone methyltransferase enhancer of zeste homologue 2. Clinical cancer research: an official journal of the American Association for Cancer Research.

[44] Zhan J, Wang P, Li S, Song J, He H, Wang Y (2019). HOXB13 networking with ABCG1/EZH2/Slug mediates metastasis and confers resistance to cisplatin in lung adenocarcinoma patients. Theranostics.

[45] Zhang S, Wang X, Osunkoya AO, Iqbal S, Wang Y, Chen Z (2011). EPLIN downregulation promotes epithelial-mesenchymal transition in prostate cancer cells and correlates with clinical lymph node metastasis. Oncogene.

[46] Zhang S, Wang X, Iqbal S, Wang Y, Osunkoya AO, Chen Z (2013). Epidermal growth factor promotes protein degradation of epithelial protein lost in neoplasm (EPLIN), a putative metastasis suppressor, during epithelial-mesenchymal transition. J Biol Chem.

[47] Wu D (2017). Epithelial protein lost in neoplasm (EPLIN): Beyond a tumor suppressor. Genes Dis.

[48] Xu J, Wang R, Xie ZH, Odero-Marah V, Pathak S, Multani A (2006). Prostate cancer metastasis: role of the host microenvironment in promoting epithelial to mesenchymal transition and increased bone and adrenal gland metastasis. Prostate.

[49] Wu D, Zhau HE, Huang WC, Iqbal S, Habib FK, Sartor O (2007). cAMP-responsive element-binding protein regulates vascular endothelial growth factor expression: implication in human prostate cancer bone metastasis. Oncogene.

[50] Thalmann GN, Anezinis PE, Chang SM, Zhau HE, Kim EE, Hopwood VL (1994). Androgen-independent cancer progression and bone metastasis in the LNCaP model of human prostate cancer. Cancer Res.

[51] Zhau HY, Chang SM, Chen BQ, Wang Y, Zhang H, Kao C (1996). Androgen-repressed phenotype in human prostate cancer. Proc Natl Acad Sci U S A.

[52] Chung LW, Zhau HE, Wu TT (1997). Development of human prostate cancer models for chemoprevention and experimental therapeutics studies. J Cell Biochem Suppl.

[53] Zhang S, Gera L, Mamouni K, Li X, Chen Z, Kucuk O (2016). Inhibition of skeletal growth of human prostate cancer by the combination of docetaxel and BKM1644: an aminobisphosphonate derivative. Oncotarget.

[54] Seo SI, Gera L, Zhau HE, Qian WP, Iqbal S, Johnson NA (2008). BKM1740, an acyl-tyrosine bisphosphonate amide derivative, inhibits the bone metastatic growth of human prostate cancer cells by inducing apoptosis. Clinical cancer research: an official journal of the American Association for Cancer Research.

[55] Gera L, Stewart J, Chung LW, Wu D Compositions and methods for treating bone cancer. 2010;US patent US2010144678-A1.

[56] Martinez Molina D, Jafari R, Ignatushchenko M, Seki T, Larsson EA, Dan C (2013). Monitoring drug target engagement in cells and tissues using the cellular thermal shift assay. Science.

[57] Axelsson H, Almqvist H, Seashore-Ludlow B, Lundback T (2020). Screening for target engagement using the cellular thermal shift assay - CETSA. In: Sittampalam GS, Grossman A, Brimacombe K, Arkin M, Auld D, Austin CP, et al. Assay guidance manual. Bethesda, Maryland: Eli Lilly & Company and the National Cancer for Advancing Translational Sciences.

[58] Seashore-Ludlow B, Axelsson H, Almqvist H, Dahlgren B, Jonsson M, Lundback T (2018). Quantitative interpretation of intracellular drug binding and kinetics using the cellular thermal shift assay. Biochemistry.

[59] Lundgren S (2019). Focusing on Relevance: CETSA-guided medicinal chemistry and lead generation. ACS Med Chem Lett.

[60] Petrilli WL, Adam GC, Erdmann RS, Abeywickrema P, Agnani V, Ai X (2020). From Screening to Targeted Degradation: Strategies for the Discovery and Optimization of Small Molecule Ligands for PCSK9. Cell Chem Biol.

[61] Comet I, Riising EM, Leblanc B, Helin K (2016). Maintaining cell identity: PRC2-mediated regulation of transcription and cancer. Nat Rev Cancer.

[62] Montgomery ND, Yee D, Chen A, Kalantry S, Chamberlain SJ, Otte AP (2005). The murine polycomb group protein Eed is required for global histone H3 lysine-27 methylation. Curr Biol.

[63] Pasini D, Bracken AP, Jensen MR, Lazzerini Denchi E, Helin K (2004). Suz12 is essential for mouse development and for EZH2 histone methyltransferase activity. EMBO J.

[64] Wu TT, Sikes RA, Cui Q, Thalmann GN, Kao C, Murphy CF (1998). Establishing human prostate cancer cell xenografts in bone: induction of osteoblastic reaction by prostate-specific antigen-producing tumors in athymic and SCID/bg mice using LNCaP and lineage-derived metastatic sublines. Int J Cancer.

[65] Mahboubi K, Li F, Plescia J, Kirkiles-Smith NC, Mesri M, Du Y (2001). Interleukin-11 up-regulates survivin expression in endothelial cells through a signal transducer and activator of transcription-3 pathway. Lab Invest.

[66] Huang H, Zhao W, Yang D (2012). Stat3 induces oncogenic Skp2 expression in human cervical carcinoma cells. Biochem Biophys Res Commun.

[67] Chen Y, Gera L, Zhang S, Li X, Yang Y, Mamouni K (2019). Small molecule BKM1972 inhibits human prostate cancer growth and overcomes docetaxel resistance in intraosseous models. Cancer Lett.

[68] Carrano AC, Eytan E, Hershko A, Pagano M (1999). SKP2 is required for ubiquitin-mediated degradation of the CDK inhibitor p27. Nat Cell Biol.

[69] Di L, Kerns E (2016). Drug-like properties: concepts, structure design, and methods from ADME to toxicity optimization. Second Edition. London, United Kingdom: Academic Press.

[70] Shou M, Martinet M, Korzekwa KR, Krausz KW, Gonzalez FJ, Gelboin HV (1998). Role of human cytochrome P450 3A4 and 3A5 in the metabolism of taxotere and its derivatives: enzyme specificity, interindividual distribution and metabolic contribution in human liver. Pharmacogenetics.

[71] Broccatelli F, Carosati E, Neri A, Frosini M, Goracci L, Oprea TI (2011). A novel approach for predicting P-glycoprotein (ABCB1) inhibition using molecular interaction fields. J Med Chem.

[72] Benet LZ (2009). The drug transporter-metabolism alliance: uncovering and defining the interplay. Mol Pharm.

[73] Grover A, Benet LZ (2009). Effects of drug transporters on volume of distribution. AAPS J.

[74] Vermeulen NP (2003). Prediction of drug metabolism: the case of cytochrome P450 2D6. Curr Top Med Chem.

[75] Zhang J, Lu Y, Dai J, Yao Z, Kitazawa R, Kitazawa S (2004). *In vivo* real-time imaging of TGF-beta-induced transcriptional activation of the RANK ligand gene promoter in intraosseous prostate cancer. Prostate.

[76] Li Y, Malaeb BS, Li ZZ, Thompson MG, Chen Z, Corey DR (2010). Telomerase enzyme inhibition (TEI) and cytolytic therapy in the management of androgen independent osseous metastatic prostate cancer. Prostate.

[77] Liang W, Wang F, Chen Q, Dai J, Escara-Wilke J, Keller ET (2019). Targeting cathepsin K diminishes prostate cancer establishment and growth in murine bone. J Cancer Res Clin Oncol.

[78] Koreckij TD, Trauger RJ, Montgomery RB, Pitts TE, Coleman I, Nguyen H (2009). HE3235 inhibits growth of castration-resistant prostate cancer. Neoplasia.

[79] Savore C, Zhang C, Muir C, Liu R, Wyrwa J, Shu J (2005). Perlecan knockdown in metastatic prostate cancer cells reduces heparin-binding growth factor responses *in vitro* and tumor growth *in vivo*. Clin Exp Metastasis.

[80] Corey E, Quinn JE, Buhler KR, Nelson PS, Macoska JA, True LD (2003). LuCaP 35: a new model of prostate cancer progression to androgen independence. Prostate.

[81] Lam HM, McMullin R, Nguyen HM, Coleman I, Gormley M, Gulati R (2017). Characterization of an Abiraterone Ultraresponsive Phenotype in Castration-Resistant Prostate Cancer Patient-Derived Xenografts. Clin Cancer Res.

[82] Suominen MI, Fagerlund KM, Rissanen JP, Konkol YM, Morko JP, Peng Z (2017). Radium-223 Inhibits Osseous Prostate Cancer Growth by Dual Targeting of Cancer Cells and Bone Microenvironment in Mouse Models. Clin Cancer Res.

[83] Kiefer JA, Vessella RL, Quinn JE, Odman AM, Zhang J, Keller ET (2004). The effect of osteoprotegerin administration on the intra-tibial growth of the osteoblastic LuCaP 23.1 prostate cancer xenograft. Clin Exp Metastasis.

[84] Nguyen HM, Vessella RL, Morrissey C, Brown LG, Coleman IM, Higano CS (2017). LuCaP Prostate Cancer Patient-Derived Xenografts Reflect the Molecular Heterogeneity of Advanced Disease an-d Serve as Models for Evaluating Cancer Therapeutics. Prostate.

[85] Shen X, Liu Y, Hsu YJ, Fujiwara Y, Kim J, Mao X (2008). EZH1 mediates methylation on histone H3 lysine 27 and complements EZH2 in maintaining stem cell identity and executing pluripotency. Mol Cell.

[86] Wassef M, Luscan A, Aflaki S, Zielinski D, Jansen P, Baymaz HI (2019). EZH1/2 function mostly within canonical PRC2 and exhibit proliferation-dependent redundancy that shapes mutational signatures in cancer. Proc Natl Acad Sci U S A.

[87] Rizq O, Mimura N, Oshima M, Saraya A, Koide S, Kato Y (2017). Dual Inhibition of EZH2 and EZH1 Sensitizes PRC2-Dependent Tumors to Proteasome Inhibition. Clin Cancer Res.

[88] Hooshfar S, Linzey MR, Wu D, Gera L, Mamouni K, Li X (2018). Sensitive liquid chromatography/tandem mass spectrometry method for the determination of two novel highly lipophilic anti-cancer drug candidates in rat plasma and tissues. Biomed Chromatogr.

[89] Li L, Feng L, Shi M, Zeng J, Chen Z, Zhong L (2017). Split luciferase-based biosensors for characterizing EED binders. Anal Biochem.

[90] H.M (2000). Berman JW, Z. Feng, G. Gilliland, T.N. Bhat, H. Weissig, I.N. Shindyalov, P.E. Bourne. The Protein Data Bank. Nucleic Acids Res.

[91] Xie ZR, Liu CK, Hsiao FC, Yao A, Hwang MJ (2013). LISE: a server using ligand-interacting and site-enriched protein triangles for prediction of ligand-binding sites. Nucleic acids research.

[92] Bello D, Webber MM, Kleinman HK, Wartinger DD, Rhim JS (1997). Androgen responsive adult human prostatic epithelial cell lines immortalized by human papillomavirus 18. Carcinogenesis.

[93] Hayward SW, Dahiya R, Cunha GR, Bartek J, Deshpande N, Narayan P (1995). Establishment and characterization of an immortalized but non-transformed human prostate epithelial cell line: BPH-1. *In vitro* Cell Dev Biol Anim.

