# Supplementary material for: Pharmacological inhibition of noncanonical EED-EZH2 signaling overcomes chemoresistance in prostate cancer
Source: Theranostics. 2021 May 8;11(14):6873–90. doi: 10.7150/thno.49235 (PMC8171087; doi:10.7150/thno.49235)
Supplement: Supplementary file 1 — Supplementary figures and tables. [file thnov11p6873s1.pdf]

Supplementary Materials and Methods

Apoptosis and Cell Cycle Analyses

For apoptosis assay, PCa cells treated with 100% DMSO or LG1980 were stained with a PE Annexin V Apoptosis Detection Kit (BioLegend, San Diego, CA) following the manufacturer's instruction. For cell cycle assay, PCa cells were stained with propidium iodide (50 μg/mL) at 37ºC for 2 h. Apoptosis and cell cycle were measured by flow cytometry with FACSCanto II flow cytometer (BD Biosciences, Bedford, MA) and results were analyzed using FlowJo software (Tree Star, Inc., Ashland, OR).

Transfection

Myc-DDK-tagged-human SKP2 expression plasmid (RC214001) and control vector pCMV6-AC-GFP were purchased from Origene (Rockville, MD). Small-interfering RNAs (siRNA) against human EZH2, Stat3, SKP2 and ABCB1 and negative control siRNAs were obtained from Santa Cruz Biotechnology, Inc (Santa Cruz, CA). Lipofectamine 2000 or Lipofectamine RNAiMAX (Life Technologies) were used for cDNA or siRNA transfection, respectively, following the manufacturer's instructions.

Real-Time PCR

Total RNA was isolated using Qiagen RNeasy Kit (Valencia, CA). cDNA was synthesized using SuperScript® III First-Strand Synthesis System (Life Technologies). Real-time PCR was performed by the Stratagene Mx3005P system (Agilent technologies) using *Power*SYBR® Green PCR Master Mix (Thermo Fisher Scientific) following the manufacturer's instruction. The primers used for real-time PCR are listed in Table S2.

Western Blot Analysis

Total cell lysates were prepared using radioimmunoprecipitation (RIPA) buffer (Santa Cruz Biotechnology). Immunoblotting analysis followed a standard procedure. Antibodies used in Western blotting are listed in Table S3.

Immunoprecipitation

Immunoprecipitation was performed using Immunoprecipitation Starter Pack (GE Healthcare Bio-Sciences Corp., Piscataway, NJ) following the manual. Antibodies used in the immunoprecipitation are listed in Table S3.

Protein Half-Life ($T_{1/2}$) Determination

PCa cells were incubated with 50μg/ml of cycloheximide (CHX, Sigma-Aldrich) for 2 h prior to the addition of LG1980 or DMSO for different times. Cell lysates were harvested for western blotting analysis. The desired protein bands from the Western blots were quantitated and normalized by the intensity of the corresponding β-actin controls using the ImageJ program. Protein degradation rate is expressed as $T_{½}$, the time for degradation of 50% of the protein, which is determined by an exponential decay fitting algorithm.

Cellular Thermal Shift Assay (CETSA)

CETSA was performed following a modified procedure as described previously [1, 2]. C4-2B-TaxR cells were incubated for 1 h in the presence of 100% DMSO, LG1980 (50 μM), or MAK683 (10 μM). A MyCycler™ thermal cycler system with gradient option (Bio-Rad Laboratories, Hercules, CA) was used for the incubation of total cell lysates at varying temperatures.

Cellular Uptake of Fluorescent Dye-Conjugated Paclitaxel

C4-2B-TaxR cells were treated with DMSO or LG1980 for 72 h and further incubated with paclitaxel-Oregon™ green 488 (Life Technologies) at 37°C for different times. Drug accumulation in live cells was recorded with a BZ-X710 fluorescence microscope (Keyence Corp, Itasca, IL).

Cytochrome P450 (CYP450) Enzyme Activity Assay

CYP450 3A4 (CYP3A4) or 2D6 (CYP2D6) enzyme inhibition was measured using P450-Glo™ Screening Systems (Promega). The CYP reactions were performed by incubating a luminogenic CYP substrate with CYP3A4 enzyme (P2377, ThermoFisher) or CYP2D6 enzyme (P2283, ThermoFisher) and NADPH

regeneration system (#V9510, Promega). The luminogenic P450-Glo™ substrates are derivatives of beetle luciferin, a substrate of firefly luciferase. The P450-Glo™ substrates (Luciferin-BE for CYP3A4 or Luciferin-ME EGE for CYP2D6) were converted by CYP enzymes to a luciferin product that in turn reacts with a Luciferin Detection Reagent to produce light. CYP3A4 and CYP2D6 reactions were performed according to the manufacturer's protocol. Briefly, a standard curve of CYP-P450 activity was prepared using a 2 mM luciferin stock solution (#E1601, Promega). To perform the test compound assay, 4 X concentrations of LG1980 along with 4 X D-luciferin standard were added into a 96-well white opaque plate. Then, 4 X CYP3A4 or CYP2D6 reaction mixture were added to the wells. After an incubation of 10 minutes at 37°C, reactions were then initiated by adding the NADPH regeneration system and incubated for 30 minutes at 37°C. At the end of the incubations, an equal volume of the luciferin detection reagent was added and luminescence was read 20 minutes later on a Cytation 5 microplate luminometer (BioTek, Winooski, VT).

***In Vivo* Toxicity**

Female CD-1 mice (5-week old, Envigo RMS, Inc, Indianapolis, IN) were randomly divided into 3 groups and received subcutaneous injection of vehicle (100% DMSO) or LG1980 at two doses (50 or 100 mg/kg), three times per week, for 2 weeks. Body weights were recorded three times per week, animal behaviors were closely observed. At the end point, major organs were examined for any abnormality, liver and kidneys were harvested and weighted.

Immunohistochemistry (IHC)

IHC expression of p-EZH2(S21) in C4-2B-TaxR xenograft specimens were performed following standard procedures [3] using a p-EZH2(S21) antibody (cat# 00388) from Bethyl Laboratories, Inc (Montgomery, TX).

Supplementary References

1. Jafari R, Almqvist H, Axelsson H, Ignatushchenko M, Lundback T, Nordlund P, et al. The cellular thermal shift assay for evaluating drug target interactions in cells. Nat Protoc. 2014; 9: 2100-22.

2. Martinez Molina D, Jafari R, Ignatushchenko M, Seki T, Larsson EA, Dan C, et al. Monitoring drug target engagement in cells and tissues using the cellular thermal shift assay. Science. 2013; 341: 84-7.

3. Chen Y, Gera L, Zhang S, Li X, Yang Y, Mamouni K, et al. Small molecule BKM1972 inhibits human prostate cancer growth and overcomes docetaxel resistance in intraosseous models. Cancer Lett. 2019; 446: 62-72.

Supplementary Tables

Table S1. Average weights of mouse organs following the treatment with vehicle or LG1980 at the indicated doses

**Treatment**	**Liver (g)**	**Kidney (g)**
**Ctrl**	2.01 ± 0.09	0.23 ± 0.04
**LG1980 50 mg/kg**	2.11 ± 0.05	0.25 ± 0.02
**LG1980 100 mg/kg**	2.01 ± 0.18	0.24 ± 0.01

Table S2. Sequences of PCR primers

**Gene**	**Forward Primer**	**Reverse Primer**
ABCB1	ACCAGATAAAAGAGAGGTGCA-ACGG	TCCCGGCCCGGATTGACTGA
CASP1	GCTATCAGGAGGGTCTTCTGG	CGCTGTACCCCAGATTTTGT
EZH2	AGTGTGACCCTGACCTCTGT	AGATGGTGCCAGCAATAGAT
GAPDH	CGAGATCCCTCCAAAATCAA	TTCACACCCATGACGAACAT
RTP4	ACATGGACGCTGAAGTTGGAT	TACGTGTGGCACAGAATCTGC
Survivin	TGCCCCGACGTTGCC	CAGTTCTTGAATGTAGAGATGCGGT

Table S3. Antibodies

Antibody	Catalog Number	Source
**ABCB1**	sc-13131	Santa Cruz Biotechnology
**β-actin**	4970	Cell Signaling Technology (Danvers, MA)
**Caspase-3**	9662	Cell Signaling Technology
**Cleaved caspase-3**	9664	Cell Signaling Technology
**E-cadherin**	3195	Cell Signaling Technology
**EED**	sc-293203	Santa Cruz Biotechnology
**EPLIN**	NB100-2305	Novus Biologicals (Littleton, CO)
**EZH1**	42088	Cell Signaling Technology
**EZH2 (for IP)**	612667	BD Transduction Laboratories (San Jose, CA)
**EZH2 (for Western blot analysis)**	5246	Cell Signaling Technology
**p-EZH2**	00388	Bethyl Laboratories, Inc (Montgomery, TX)
**GAPDH**	sc-20357	Santa Cruz Biotechnology
**H3 Histone**	07-690	Millipore (Burlington, MA)
**Mono-methyl-histone H3 lysine**	07-448	Millipore
**Di-methyl-histone H3 lysine**	9728	Cell Signaling Technology
**Tri-methyl-histone H3 lysine**	9733	Cell Signaling Technology
**Methyl Lysine**	14680	Cell Signaling Technology
**Normal mouse IgG**	sc-2025	Santa Cruz Biotechnology
**Normal rabbit IgG**	011-000-003	Jackson ImmunoResearch Laboratories, Inc (West Grove, PA)
**p21**	556430	BD Medical Technology

**p27**	2552	Cell Signaling Technology
**PARP**	9542	Cell Signaling Technology
**Cleaved PARP**	5625	Cell Signaling Technology
**SKP2**	32-3300	Life Technologies
**Stat3**	4904	Cell Signaling Technology
**p-Stat3(Ser727)**	9134	Cell Signaling Technology
**Survivin**	NB500-201	Novus Biologicals (Littleton, CO)
**SUZ12**	sc-271325	Santa Cruz Biotechnology
**Ubiquitin**	BML-PW8810	Enzo Life Sciences, Inc (Ann Arbor, MI)

Supplementary Figures

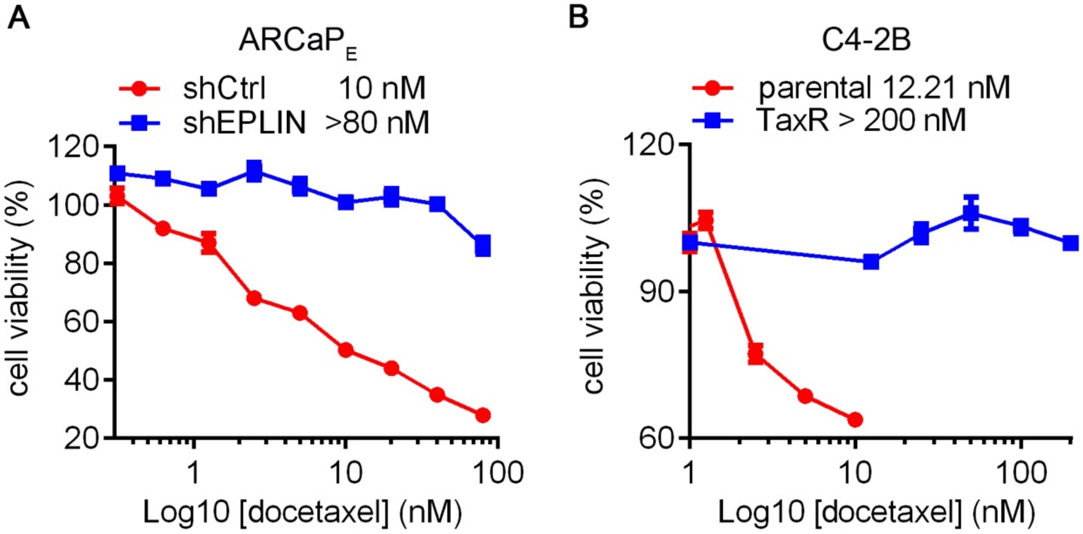

Figure S1. *In vitro* cytotoxicity of docetaxel in the $ARCaP_E$ (A) and C4-2B (B) models (72 h).

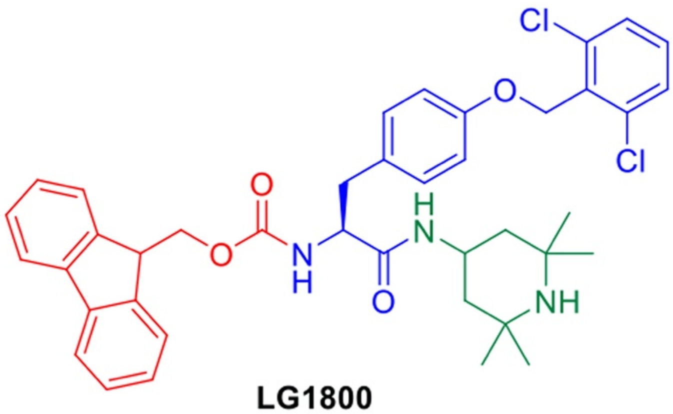

LG1800

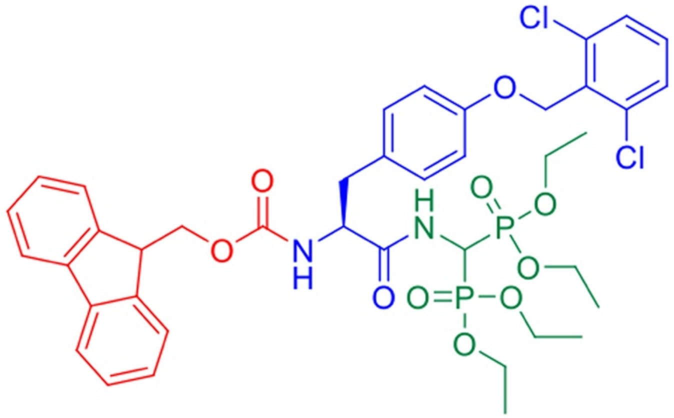

LG2134

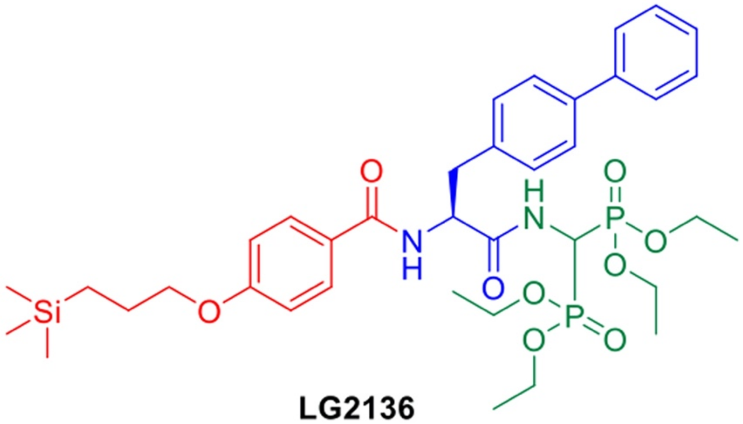

LG2136

Figure S2. Chemical structures of several LG1980 analogues.

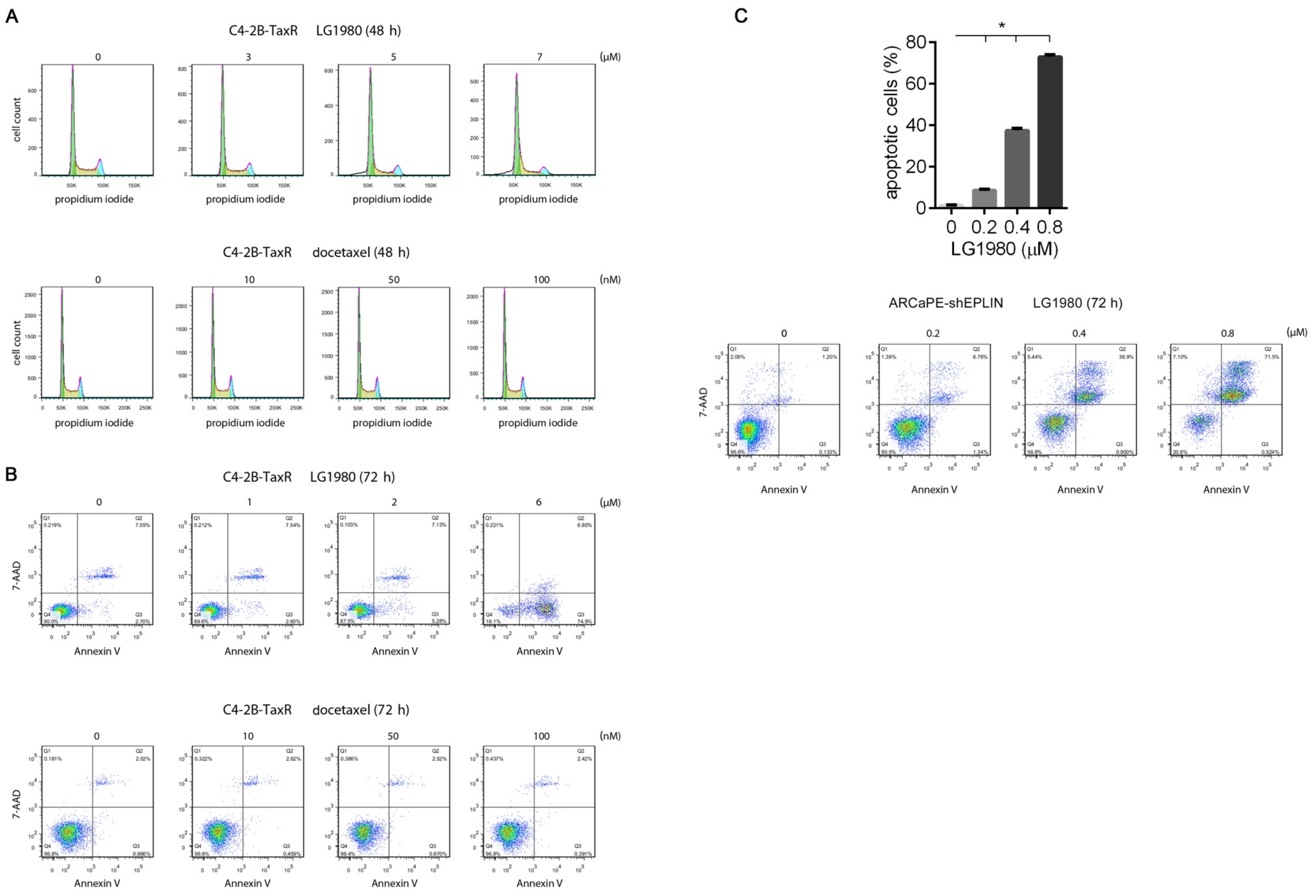
A
C4-2B-TaxR LG1980 (48 h)
0
3
5
7
(μM)
cell count
propidium iodide
C4-2B-TaxR docetaxel (48 h)
0
10
50
100
(nM)
B
C4-2B-TaxR LG1980 (72 h)
0
1
2
6
(μM)
7-AAD
Annexin V
C4-2B-TaxR docetaxel (72 h)
0
10
50
100
(nM)
C
apoptotic cells (%)
LG1980 (μM)
*
ARCaPE-shEPLIN LG1980 (72 h)
0
0.2
0.4
0.8
(μM)

Figure S3. (A) Original flow cytometry results on cell cycle in C4-2B-TaxR cells following LG1980 or docetaxel treatment at the indicated concentrations (48 h). (B) Original flow cytometry results on Annexin V expression in C4-2B-TaxR cells following LG1980 or docetaxel treatment at the indicated concentrations (72 h). (C) Upper: Flow cytometry analysis on Annexin V staining in ARCaP$_E$-shEPLIN cells following LG1980 treatment at the indicated concentrations (72 h). *$p < 0.05$; Bottom: Original flow cytometry results on Annexin V staining in ARCaP$_E$-shEPLIN cells following LG1980 treatment at the indicated concentrations (72 h).

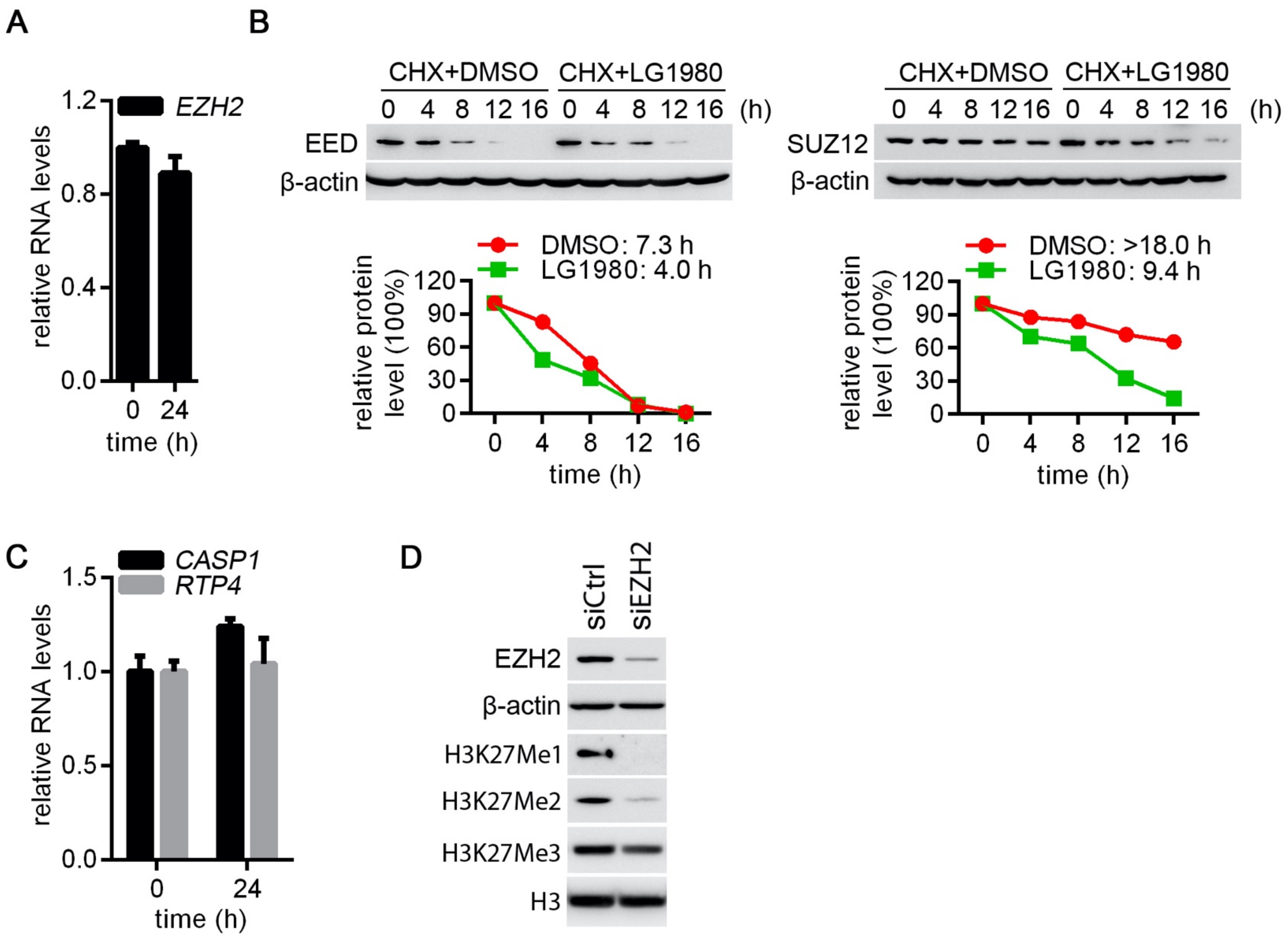

Figure S4. (A) qPCR analysis of RNA expression of EZH2 in C4-2B-TaxR cells treated with LG1980 (7 µM, 24 h). (B) Upper: Expression of EED and SUZ12 in C4-2B-TaxR cells treated with DMSO or LG1980 (7 µM) in the presence of CHX; Bottom: Calculated half-life of EED or SUZ12 protein in C4-2B-TaxR cells treated with DMSO or LG1980 (7 µM). (C) qPCR analysis of RNA expression of CASP1 and RPT4 in C4-2B-TaxR cells treated with LG1980 (7 µM, 24 h). (D) Western blot analysis of H3K27 methylation in C4-2B-TaxR cells transfected with EZH2 siRNA (60 µM) or control siRNA (72 h).

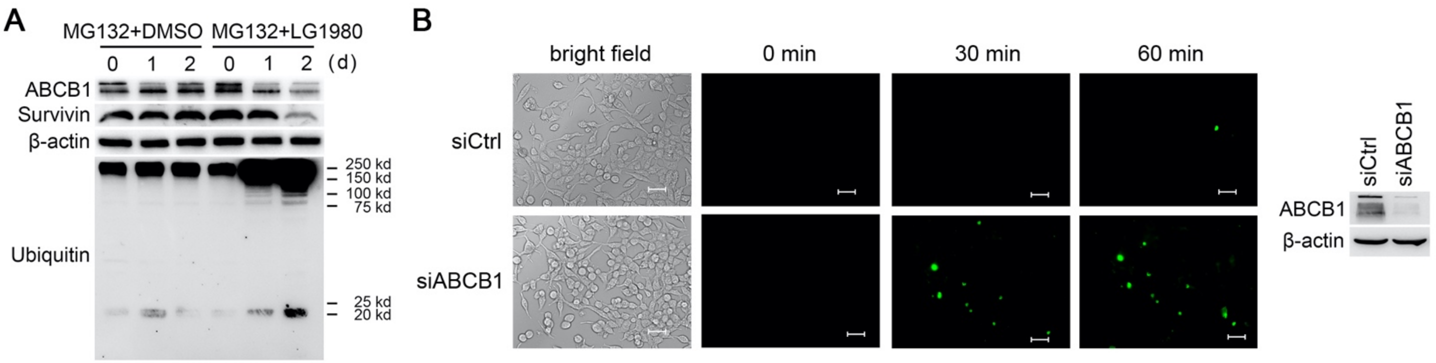

Figure S5. (A) Western blot analysis of protein expression of ABCB1, survivin and ubiquitin in C42B-TaxR cells pre-treated with MG132 (20 μM for 3 h, then 2 μM for 24 or 48 h) and DMSO or LG1980 (7 μM) for the indicated times. (B) Left: Fluorescence microscopy images of cellular uptake of Oregon Green 488-paclitaxel in C4-2B-TaxR cells. The cells were transfected with ABCB1 siRNA (30 nM) or control siRNA for 72 h prior to paclitaxel incubation for the indicated times. Scale bar: 50 μm; Right: Western blot analysis of ABCB1 expression in C4-2B-TaxR cells transfected with control siRNA or ABCB1 siRNA.

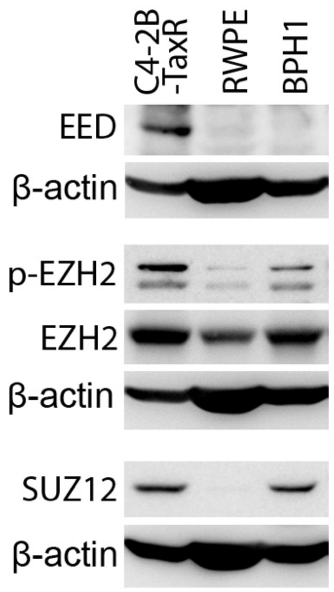

Figure S6. Western blot analysis of basal expression of core PRC2 components in C4-2B-TaxR, RWPE-1 and BPH1 cells.

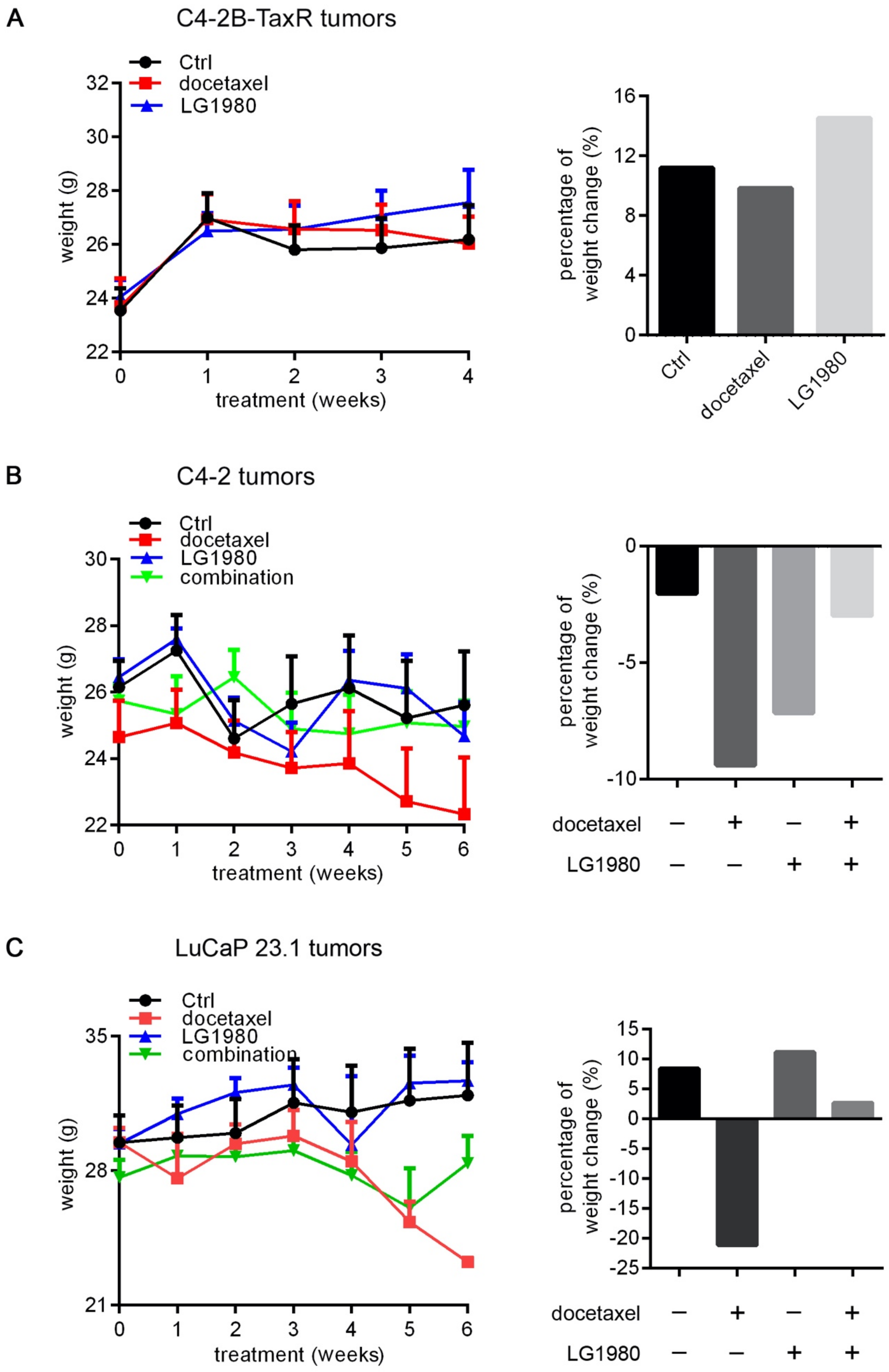
A
C4-2B-TaxR tumors
Ctrl
docetaxel
LG1980
weight (g)
treatment (weeks)
percentage of weight change (%)
Ctrl
docetaxel
LG1980
B
C4-2 tumors
Ctrl
docetaxel
LG1980
combination
weight (g)
treatment (weeks)
percentage of weight change (%)
docetaxel
LG1980
C
LuCaP 23.1 tumors
Ctrl
docetaxel
LG1980
combination
weight (g)
treatment (weeks)
percentage of weight change (%)
docetaxel
LG1980

Figure S7. Effect of treatments on mouse body weight (A) Left: Average body weights of C4-2B-TaxR tumor-bearing mice in different treatment groups. Right: percentage of mouse body weight change in different treatment groups. (B) Left: Average body weights of C4-2 tumor-bearing mice in different treatment groups. Right: percentage of mouse body weight change in different treatment groups. (C) Left: Average body weights of LuCaP 23.1 tumor-bearing mice in different treatment groups. Right: percentage of mouse body weight change in different treatment groups.

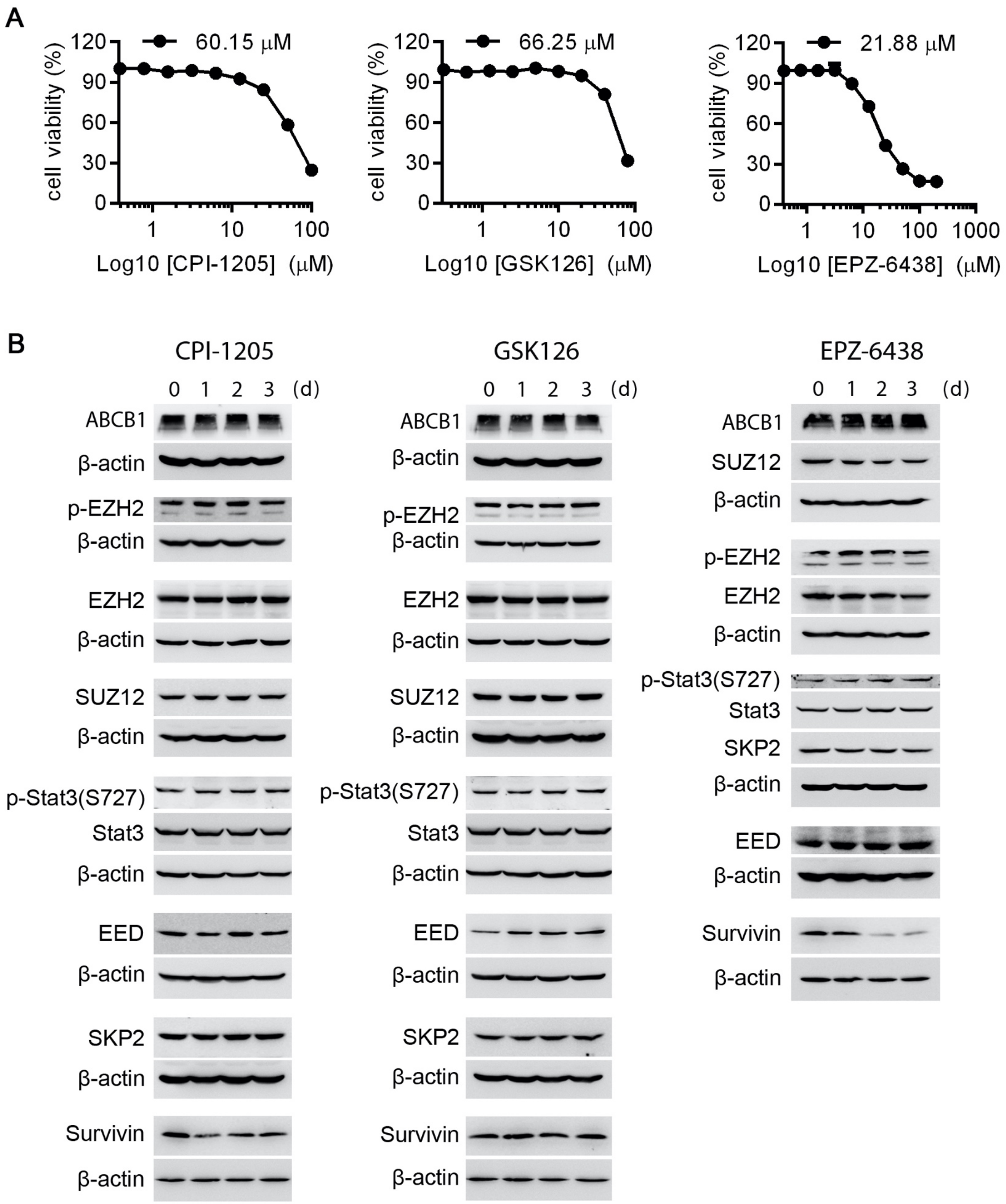
A
60.15 μM
66.25 μM
21.88 μM
cell viability (%)
120
90
60
30
0
1
10
100
1000
Log10 [CPI-1205] (μM)
Log10 [GSK126] (μM)
Log10 [EPZ-6438] (μM)
B
CPI-1205
GSK126
EPZ-6438
0 1 2 3 (d)
ABCB1
β-actin
p-EZH2
EZH2
SUZ12
p-Stat3(S727)
Stat3
EED
SKP2
Survivin

Figure S8. (A) *In vitro* cytotoxicity of three known EZH2 inhibitors (GSK126, CPI-1205 and EPZ-6438) in C4-2B-TaxR cells (72 h). (B) Western blot analysis of putative noncanonical EZH2 signaling components in C4-2B-TaxR cells treated with three known EZH2 inhibitors (GSK126, CPI-1205 and EPZ-6438).

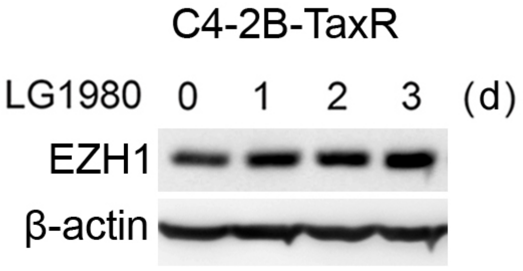

Figure S9. Western blot analysis of EZH1 expression in C4-2B-TaxR cells treated with LG1980 (7 μM) at the indicated time points.

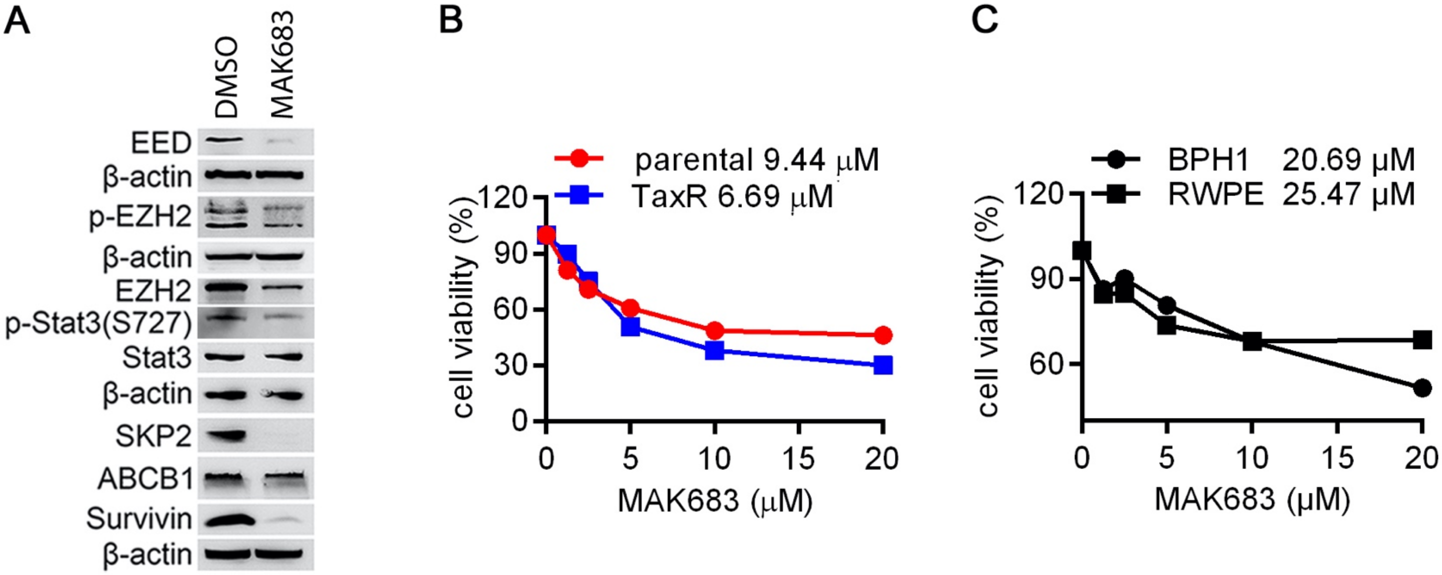

Figure S10. (A) Western blot analysis of the effects of MAK683 on EZH2 signaling in C4-2B-TaxR cells (7 μM, 72h). (B) *In vitro* cytotoxicity of MAK683 in C4-2B and C4-2B-TaxR cells (72 h). (C) CCK-8 assay of the *in vitro* cytotoxicity of MAK683 in BPH-1 and RWPE-1 cells (72 h).

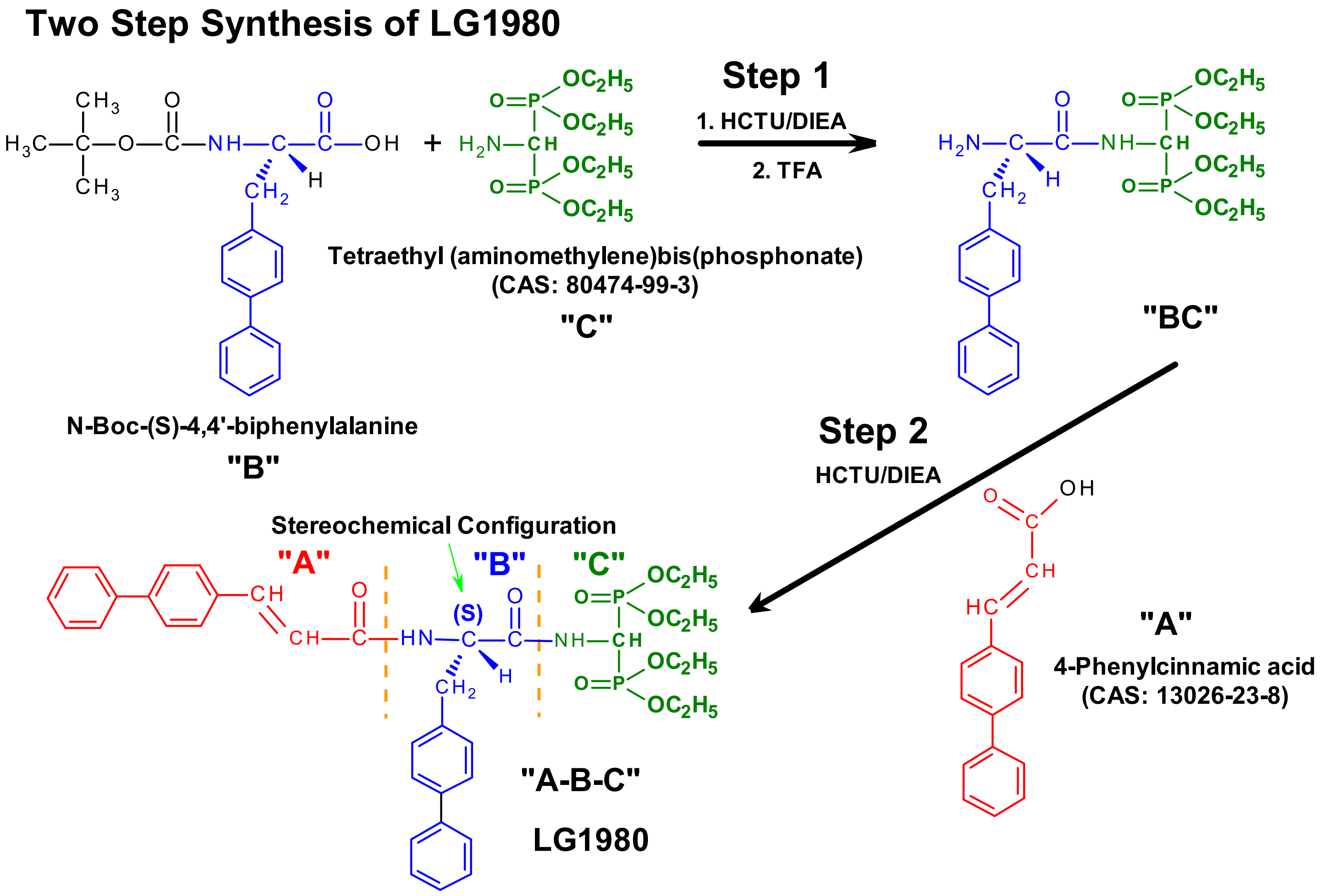

Figure S11. Two-step synthesis of LG1980.
